# Supplementary material for: Topological minibands and interaction driven quantum anomalous Hall state in topological insulator based moiré heterostructures
Source: Nat Commun. 2024 Mar 26;15:2670. doi: 10.1038/s41467-024-46717-7 (PMC11258263; doi:10.1038/s41467-024-46717-7)
Supplement: Supplementary file 2 — Supplementary Information [file 41467_2024_46717_MOESM2_ESM.pdf]

Supplementary Information for "Topological Minibands and Interaction Driven Quantum Anomalous Hall State in Topological Insulator Based Moiré Heterostructures"

Supplementary Note 1. TOPOLOGY OF THE LOWEST CONDUCTION BANDS

A. Perturbation Theory and emergent chiral symmetry

In this section, the topology of inversion symmetric moiré system in Fig. 2(a) of the main text with $\phi = 0, \alpha = 1, V_0/E_0 = 0$ is studied under the perturbation of moiré potential strength Δ_1.

By the Fu-Kane parity criterion[1], the $\mathbb{Z}_2$ invariant ν can be determined by the parities λ_i at time reversal ($\mathcal{T}$) invariant momenta one Γ and three M of moiré Brillouin Zone (MBZ) for one of the degenerate states by $(-1)^\nu = \prod_i \lambda_i$ At $\phi = 0, \alpha = 1, V_0/E_0 = 0$, the crystal symmetry of this system is described by the point group D_{6h} with six-fold rotation C_{6z} about the z-axis, the inversion $\mathcal{I}$, the y-directional mirror $\mathcal{M}_y$ and the z-directional mirror $\mathcal{M}_z$. We label the original basis of our model Hamiltonian Eq. 1 in main text by $|\mathbf{k}, J_z, \alpha\rangle$, where $\mathbf{k}$ is the momentum, $J_z = \pm 1/2$ labels two spin states of surface states and $\alpha = \mathrm{t}, \mathrm{b}$ labels the top and bottom surfaces. The z-directional mirror $\mathcal{M}_z$ transforms the top surface to the bottom surface and thus it relates the basis wave-functions on two surfaces by

$$\mathcal{M}_z|\mathbf{k}, J_z, \mathrm{t}\rangle = e^{-i\pi J_z}|\mathbf{k}, J_z, \mathrm{b}\rangle \quad \mathcal{M}_z|\mathbf{k}, J_z, \mathrm{b}\rangle = e^{-i\pi J_z}|\mathbf{k}, J_z, \mathrm{t}\rangle. \tag{1}$$

We may transform the basis wave-functions to the bonding and anti-bonding states of two surface states as

$$|\mathbf{k}, J_z, I\rangle = \frac{1}{\sqrt{2}}(|\mathbf{k}, J_z, \mathrm{t}\rangle + I|\mathbf{k}, J_z, \mathrm{b}\rangle) \tag{2}$$

with $I = \pm$ labels the transformation property under the inversion parity

$$\mathcal{I}|\mathbf{k}, J_z, \pm\rangle = \pm|-\mathbf{k}, J_z, \pm\rangle \tag{3}$$

and the eigen-values of the $\mathcal{M}_z$ operator

$$\mathcal{M}_z|\mathbf{k}, J_z, \pm\rangle = \pm e^{-i\pi J_z}|\mathbf{k}, J_z, \pm\rangle. \tag{4}$$

On these bonding and anti-bonding basis

$$|\mathbf{k}, J_z, I\rangle = |\mathbf{k}, +\frac{1}{2}, +\rangle, |\mathbf{k}, -\frac{1}{2}, -\rangle, |\mathbf{k}, -\frac{1}{2}, +\rangle, |\mathbf{k}, +\frac{1}{2}, -\rangle, \tag{5}$$

the Hamiltonian H_0 in the main text Eq.1 can be written in a block diagonal form,

$$H_0(\mathbf{r}) = H^{\mathrm{TI}} + H^{\mathrm{M}}(\mathbf{r}) = \begin{pmatrix} H^{\mathrm{TI}}_{m_z=-i} & 0 \\ 0 & H^{\mathrm{TI}}_{m_z=+i} \end{pmatrix} + \Delta(\mathbf{r}) I_{4\times4}. \tag{6}$$

with

$$H^{\mathrm{TI}}_{m_z=\pm i} = \begin{pmatrix} m & \mp iv\hat{k}_\pm \\ \pm iv\hat{k}_\mp & -m \end{pmatrix}, \tag{7}$$

where $\hat{k}_\pm = -i(\partial_x \pm i\partial_y)$, $m = m_0 + m_2(-\partial_x^2 - \partial_y^2)$, $I_{4\times4}$ is a 4×4 identity matrix, and $m_z = \pm i$ labels the eigen-values of the mirror operator $\mathcal{M}_z$. H^{M} is the moiré potential with $\phi = 0, \alpha = 1, V_0/E_0 = 0$.

We next determine the parities of lower-energy minibands at $\mathcal{T}$ invariant momenta, including one Γ and three M in the moiré BZ, of the Hamiltonian H_0 in the limit $|\Delta_1| \ll |v\mathbf{b}_1^{\mathrm{M}}|, |m|$ via perturbation theory. As the H_0 is block diagonal in the $m_z = \pm i$ subspace, we may perform the perturbation calculation for the $m_z = -i$ block while the miniband parity of the $m_z = +i$ block can be related by TR symmetry. For the $m_z = -i$ block, the unperturbed Hamiltonian is $H^{\mathrm{TI}}_{m_z=-i}$ while H^{M} is treated as the perturbation. We choose the eigen-wavefunctions of $H^{\mathrm{TI}}_{m_z=-i}$ to possess a well-defined gauge at Γ in the moiré BZ, which can be written as

$$|\psi^{\mathrm{TI}}_{+,-i}(\mathbf{k})\rangle = \begin{pmatrix} i\cos\frac{\theta_\mathbf{k}}{2} \\ \sin\frac{\theta_\mathbf{k}}{2}e^{i\phi_\mathbf{k}} \end{pmatrix} \quad |\psi^{\mathrm{TI}}_{-,-i}(\mathbf{k})\rangle = \begin{pmatrix} -i\sin\frac{\theta_k}{2}e^{-i\phi_k} \\ \cos\frac{\theta_k}{2} \end{pmatrix} \tag{8}$$

for $m > 0$ with the eigen-energies $E_{\pm}^{\rm TI}(\mathbf{k}) = \pm\sqrt{m^2+v^2k^2}$ and

$$|\psi_{+,-i}^{\rm TI}(\mathbf{k})\rangle = \begin{pmatrix} i\sin\frac{\theta_k}{2} \\ -\cos\frac{\theta_k}{2}e^{i\phi_k} \end{pmatrix} \quad |\psi_{-,-i}^{\rm TI}(\mathbf{k})\rangle = \begin{pmatrix} i\cos\frac{\theta_{\mathbf{k}}}{2}e^{-i\phi_{\mathbf{k}}} \\ \sin\frac{\theta_{\mathbf{k}}}{2} \end{pmatrix} \tag{9}$$

for $m < 0$ with the eigen-energies $E_{\pm}^{\rm TI}(\mathbf{k}) = \mp\sqrt{m^2+v^2k^2}$, where $\cos\theta_k = m/\sqrt{m^2+v^2k^2}$ and $ke^{i\phi_k} = k_x + ik_y$.

$$\mathcal{I}|\psi_{I,-i}^{\rm TI}(\mathbf{k})\rangle = I|\psi_{I,-i}^{\rm TI}(-\mathbf{k})\rangle, \tag{10}$$

and the expression for the eigen-energy can be unified as

$$E_I^{\rm TI}(\mathbf{k}) = \mathrm{sgn}(m)I\sqrt{m^2+v^2k^2}, \tag{11}$$

so the inversion parity I also labels different eigen-energies of our model Hamiltonian. The second lower-index $-i$ in the eigen-state labels the $\mathcal{M}_z$ eigen-values. This definition of the eigen-states $|\psi_{I,m_z}^{\rm TI}\rangle$ is also used in the main text. From the expression of the eigen-energies (11), the higher energy state with $E_{\rm CB}^{\rm TI}(\mathbf{k}) = E_{I=+\mathrm{sgn}(m)}^{\rm TI}(\mathbf{k})$ that corresponds to the conduction bands should be given by

$$|\psi_{{\rm CB},-i}^{\rm TI}(\mathbf{k})\rangle = |\psi_{+\mathrm{sgn}(m),-i}^{\rm TI}(\mathbf{k})\rangle \tag{12}$$

and the lower energy state with $E_{\rm VB}^{\rm TI}(\mathbf{k}) = E_{I=-\mathrm{sgn}(m)}^{\rm TI}(\mathbf{k})$ for the valence bands should be

$$|\psi_{{\rm VB},-i}^{\rm TI}(\mathbf{k})\rangle = |\psi_{-\mathrm{sgn}(m),-i}^{\rm TI}(\mathbf{k})\rangle. \tag{13}$$

For $m_z = +i$ subspace, we use TR symmetry operator, given by

$$\mathcal{T} = \begin{pmatrix} 0 & 0 & -1 & 0 \\ 0 & 0 & 0 & 1 \\ 1 & 0 & 0 & 0 \\ 0 & -1 & 0 & 0 \end{pmatrix}\mathcal{K} \tag{14}$$

in the basis Eq. (5) with $\mathcal{K}$ for complex conjugate, to define

$$|\psi_{I,+i}^{\rm TI}(\mathbf{k})\rangle = -i\mathcal{T}|\psi_{I,-i}^{\rm TI}(-\mathbf{k})\rangle, \tag{15}$$

and the commutation relation $[\mathcal{T},\mathcal{I}] = 0$ leads to the same inversion parity for two degenerate states $|\psi_{I,m_z=\pm i}^{\rm TI}(\Gamma_i)\rangle$ at any TR-invariant momentum Γ_i.

The band gap and the inversion parity of minibands at Γ of the moiré BZ are determined by the hybridization term m in the limit $|\Delta_1| \ll |m|$, for which the moiré potential does not play a role. Thus, we only need to consider the unperturbed Hamiltonian $H_{m_z=-i}^{\rm TI}$ in Eq. (7), which is diagonal, and the eigen-state $|\psi_{+,-i}^{\rm TI}(\Gamma)\rangle = (1,0)^T$ has the eigen-energy m and $|\psi_{-,-i}^{\rm TI}(\Gamma)\rangle = (0,1)^T$ has the eigen-energy $-m$. The lower index I directly gives the parity of the eigen-state at Γ, namely $\mathcal{I}|\psi_{I,-i}^{\rm TI}(\Gamma)\rangle = I|\psi_{I,-i}^{\rm TI}(\Gamma)\rangle$. The parity of the CB1 for the eigen-state $|\psi_{{\rm CB},-i}^{\rm TI}(\Gamma)\rangle$ is $\lambda_\Gamma = +\mathrm{sgn}(m)$ and that of the VB1 for the eigen-state $|\psi_{{\rm VB},-i}^{\rm TI}(\Gamma)\rangle$ is $\lambda_\Gamma = -\mathrm{sgn}(m)$, depending on the sign of m. Therefore, the parities of CB1 and VB1 at Γ are opposite,

$$\lambda_\Gamma^{\rm CB1} = -\lambda_\Gamma^{\rm VB1}. \tag{16}$$

Different from the Γ point, the moiré potential is essential in determining the parities of the minibands at M in the moiré BZ. Without moiré potential, the eigen-states $|\psi_{I,-i}^{\rm TI}(\mathbf{k})\rangle$ of $H_{m_z=-i}^{\rm TI}$ at $\mathbf{k} = \mathbf{M} = \frac{1}{2}\mathbf{b}_1^{\rm M}$ and $\mathbf{k} = \mathbf{M} - \mathbf{b}_1^{\rm M} = -\mathbf{M}$ are degenerate, so the spectrum is gapless at M, even with a finite m. The moiré potential will couple these two states at $\mathbf{M}$ and $-\mathbf{M}$ as both belong to the same momentum in moiré BZ. By projecting the full Hamiltonian $H_0(\mathbf{r})$ into the subspace spanned by these two states $|\psi_{{\rm CB},-i}^{\rm TI}(\pm\mathbf{M})\rangle$, we find the effective Hamiltonian $H_{eff}^{\rm CB}(\mathbf{M})$ for CB1 and CB2 is given through the degenerate perturbation by

$$\begin{aligned} H_{eff}^{\rm CB}(\mathbf{M}) &= \begin{pmatrix} \langle\psi_{{\rm CB},-i}^{\rm TI}(\mathbf{M})|H^{\rm TI}|\psi_{{\rm CB},-i}^{\rm TI}(\mathbf{M})\rangle & \langle\psi_{{\rm CB},-i}^{\rm TI}(\mathbf{M})|H^{\rm M}|\psi_{{\rm CB},-i}^{\rm TI}(-\mathbf{M})\rangle \\ \langle\psi_{{\rm CB},-i}^{\rm TI}(-\mathbf{M})|H^{\rm M}|\psi_{{\rm CB},-i}^{\rm TI}(\mathbf{M})\rangle & \langle\psi_{{\rm CB},-i}^{\rm TI}(-\mathbf{M})|H^{\rm TI}|\psi_{{\rm CB},-i}^{\rm TI}(-\mathbf{M})\rangle \end{pmatrix} \\ &= \begin{pmatrix} E_{\rm CB}^{\rm TI}(\mathbf{M}) & \Delta_1|\cos\theta_{k_{\rm M}}| \\ \Delta_1|\cos\theta_{k_{\rm M}}| & E_{\rm CB}^{\rm TI}(-\mathbf{M}) \end{pmatrix}, \end{aligned} \tag{17}$$

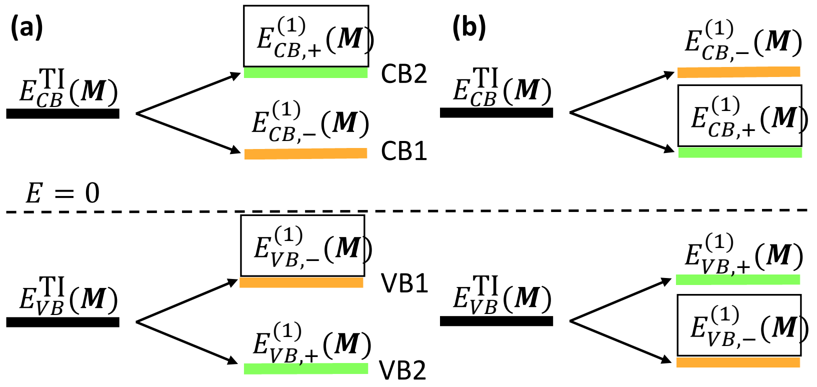

Supplementary Figure 1. Schematic figures of energies under the first order perturbation. The orange (green) lines are states with odd (even) parities. The framed/unframed energies have the first order energy perturbation related by chiral symmetries. (a) $\langle\Psi_{{\rm CB},+,m_z}(\mathbf{M})|H^{\rm M}|\Psi_{{\rm CB},+,m_z}(\mathbf{M})\rangle > \langle\Psi_{{\rm CB},-,m_z}(\mathbf{M})|H^{\rm M}|\Psi_{{\rm CB},-,m_z}(\mathbf{M})\rangle$. (b) $\langle\Psi_{{\rm CB},+,m_z}(\mathbf{M})|H^{\rm M}|\Psi_{{\rm CB},+,m_z}(\mathbf{M})\rangle < \langle\Psi_{{\rm CB},-,m_z}(\mathbf{M})|H^{\rm M}|\Psi_{{\rm CB},-,m_z}(\mathbf{M})\rangle$ for $m_z = \pm i$.

where $E^{\rm TI}_{\rm CB}(\mathbf{M}) = E^{\rm TI}_{\rm CB}(-\mathbf{M}) = \sqrt{m^2+v^2k_{\rm M}^2}, \cos\theta_{k_{\rm M}} = m/\sqrt{m^2+v^2k_{\rm M}^2}$, and $k_{\rm M} = |\mathbf{M}|$. The eigen-state $(|\psi^{\rm TI}_{{\rm CB},-i}(\mathbf{M})\rangle + |\psi^{\rm TI}_{{\rm CB},-i}(-\mathbf{M})\rangle)/\sqrt{2}$ has eigen-energy $E^{\rm TI}_{\rm CB}(\mathbf{M}) + \Delta_1|\cos\theta_{\mathbf{k}}|$ and the parity $+{\rm sgn}(m)$ while the eigen-state $(|\psi^{\rm TI}_{{\rm CB},-i}(\mathbf{M})\rangle - |\psi^{\rm TI}_{{\rm CB},-i}(-\mathbf{M})\rangle)/\sqrt{2}$ has eigen-energy $E^{\rm TI}_{\rm CB}(\mathbf{M}) - \Delta_1|\cos\theta_{\mathbf{k}}|$ and the parity $-{\rm sgn}(m)$. The lower energy state (eigen-energy $E^{\rm TI}_{\rm CB}(\mathbf{M}) - |\Delta_1||\cos\theta_{\mathbf{k}}|$), which corresponds to CB1, depends on the sign of Δ_1 and is given by

$$|\psi_{{\rm CB1},-i}(\mathbf{M})\rangle = (|\psi^{\rm TI}_{{\rm CB},-i}(\mathbf{M})\rangle - {\rm sgn}(\Delta_1)|\psi^{\rm TI}_{{\rm CB},-i}(-\mathbf{M})\rangle)/\sqrt{2} \tag{18}$$

with the parity $\lambda^{\rm CB1}_{\mathbf{M}} = (+{\rm sgn}(m))(-{\rm sgn}(\Delta_1))$. For VB1 and VB2, the effective Hamiltonian at $\mathbf{M}$ is given by

$$\begin{aligned} H^{\rm VB}_{eff}(\mathbf{M}) &= \begin{pmatrix} \langle\psi^{\rm TI}_{{\rm VB},-i}(\mathbf{M})|H^{\rm TI}|\psi^{\rm TI}_{{\rm VB},-i}(\mathbf{M})\rangle & \langle\psi^{\rm TI}_{{\rm VB},-i}(\mathbf{M})|H^{\rm M}|\psi^{\rm TI}_{{\rm VB},-i}(-\mathbf{M})\rangle \\ \langle\psi^{\rm TI}_{{\rm VB},-i}(-\mathbf{M})|H^{\rm M}|\psi^{\rm TI}_{{\rm VB},-i}(\mathbf{M})\rangle & \langle\psi^{\rm TI}_{{\rm VB},-i}(-\mathbf{M})|H^{\rm TI}|\psi^{\rm TI}_{{\rm VB},-i}(-\mathbf{M})\rangle \end{pmatrix} \\ &= \begin{pmatrix} E^{\rm TI}_{\rm VB}(\mathbf{M}) & \Delta_1|\cos\theta_{k_{\rm M}}| \\ \Delta_1|\cos\theta_{k_{\rm M}}| & E^{\rm TI}_{\rm VB}(-\mathbf{M}) \end{pmatrix}, \end{aligned} \tag{19}$$

where $E^{\rm TI}_{\rm VB}(\mathbf{M}) = -\sqrt{m^2+v^2k_{\rm M}^2}$. The eigen-state $(|\psi^{\rm TI}_{{\rm VB},-i}(\mathbf{M})\rangle + |\psi^{\rm TI}_{{\rm VB},-i}(-\mathbf{M})\rangle)/\sqrt{2}$ has eigen-energy $E^{\rm TI}_{\rm VB}(\mathbf{M}) + \Delta_1|\cos\theta_{k_{\rm M}}|$ and the parity $-{\rm sgn}(m)$ while the eigen-state $(|\psi^{\rm TI}_{{\rm VB},-i}(\mathbf{M})\rangle - |\psi^{\rm TI}_{{\rm VB},-i}(-\mathbf{M})\rangle)/\sqrt{2}$ has eigen-energy $E^{\rm TI}_{\rm VB}(\mathbf{M}) - \Delta_1|\cos\theta_{k_{\rm M}}|$ and the parity ${\rm sgn}(m)$. The higher energy state (eigen-energy $E^{\rm TI}_{\rm VB}(\mathbf{M}) + |\Delta_1||\cos\theta_{k_{\rm M}}|$), which corresponds to VB1, is given by

$$|\psi_{{\rm VB1},-i}(\mathbf{M})\rangle = (|\psi^{\rm TI}_{{\rm VB},-i}(\mathbf{M})\rangle + {\rm sgn}(\Delta_1)|\psi^{\rm TI}_{{\rm VB},-i}(-\mathbf{M})\rangle)/\sqrt{2} \tag{20}$$

with the parity $\lambda^{\rm VB1}_{\mathbf{M}} = (-{\rm sgn}(m))(+{\rm sgn}(\Delta_1))$. Thus,

$$\lambda^{\rm CB1}_{\mathbf{M}} = \lambda^{\rm VB1}_{\mathbf{M}} = -{\rm sgn}(m){\rm sgn}(\Delta_1). \tag{21}$$

The parity at $\mathbf{M}$ for CB1 and VB1 are the same. Because the $\mathbb{Z}_2$ invariant is $(-1)^\nu = \lambda_\Gamma(\lambda_{\mathbf{M}})^3$, $(-1)^{\nu_{\rm CB1}} = -{\rm sgn}(\Delta_1)$ and $(-1)^{\nu_{\rm VB1}} = +{\rm sgn}(\Delta_1)$, so that $\nu_{\rm CB1}$ and $\nu_{\rm VB1}$ are differed by 1. Thus, we conclude $\nu_{CB1} + \nu_{VB1} = 1$ mod 2, namely one of CB1 and VB1 has nonzero $\mathbb{Z}_2$ invariant and the other has trivial $\mathbb{Z}_2$ invariant.

The above conclusion of topology of CB1 and VB1 can also be understood from the chiral symmetry operator $\mathcal{C}$ of $H^{\rm TI}$, defined by $\mathcal{C} = \tau_z s_z$, when the chemical potential is at the charge neutrality point, where τ acts on the top/bottom surface degrees of freedom and s acts on spin. The emergence of the chiral symmetry requires dropping higher-order k terms, e.g. k^2 terms, in $H^{\rm TI}$, which are not important at the moiré energy scale. This operator has the commutation relations

$$\{\mathcal{C}, H^{\rm TI}\} = 0, [\mathcal{C}, H^{\rm M}] = 0, \{\mathcal{C}, \mathcal{I}\} = 0, \{\mathcal{C}, \mathcal{M}_z\} = 0. \tag{22}$$

On the basis of Eq. (5), the form of chiral symmetry operator is transformed into

$$\mathcal{C} = \begin{pmatrix} 0 & 0 & 0 & 1 \\ 0 & 0 & -1 & 0 \\ 0 & -1 & 0 & 0 \\ 1 & 0 & 0 & 0 \end{pmatrix}, \tag{23}$$

which mixes the eigen-states with opposite $\mathcal{M}_z$ eigen-values, namely

$$|\psi^{\mathrm{TI}}_{-I,+i}(\mathbf{k})\rangle = \mathcal{C}|\psi^{\mathrm{TI}}_{I,-i}(\mathbf{k})\rangle. \tag{24}$$

This implies

$$|\psi_{\mathrm{VB},+i}(\mathbf{k})\rangle = \mathcal{C}|\psi_{\mathrm{CB},-i}(\mathbf{k})\rangle = \mathcal{C}|\psi^{\mathrm{TI}}_{+\mathrm{sgn}(m),-i}(\mathbf{k})\rangle = |\psi^{\mathrm{TI}}_{-\mathrm{sgn}(m),+i}(\mathbf{k})\rangle. \tag{25}$$

At Γ, the opposite parities between $|\psi_{\mathrm{CB},-i}(\Gamma)\rangle$ and $|\psi_{\mathrm{VB},+i}(\Gamma)\rangle$ ($+\mathrm{sgn}(m)$ for $|\psi_{\mathrm{CB},-i}(\Gamma)\rangle$ and $-\mathrm{sgn}(m)$ for $|\psi_{\mathrm{VB},+i}(\Gamma)\rangle$) directly come from the anti-commutation relation $\{\mathcal{C},\mathcal{I}\} = 0$.

At M, the CB1 (VB1) and CB2 (VB2) are degenerate for H^{TI}, so we need to consider the first order perturbation from H^{M}. For the convenience of the discussion, we introduce the inversion adapted basis functions for CB1, CB2, VB1, and VB2 as

$$\begin{aligned} |\Psi_{\mathrm{CB},I,m_z}(\mathbf{M})\rangle &= \frac{1}{\sqrt{2}}\left(|\psi^{\mathrm{TI}}_{\mathrm{CB},m_z}(\mathbf{M})\rangle + I\mathrm{sgn}(m)|\psi^{\mathrm{TI}}_{\mathrm{CB},m_z}(-\mathbf{M})\rangle\right) \\ |\Psi_{\mathrm{VB},I,m_z}(\mathbf{M})\rangle &= \frac{1}{\sqrt{2}}\left(|\psi^{\mathrm{TI}}_{\mathrm{VB},m_z}(\mathbf{M})\rangle - I\mathrm{sgn}(m)|\psi^{\mathrm{TI}}_{\mathrm{VB},m_z}(-\mathbf{M})\rangle\right) \end{aligned} \tag{26}$$

with the parity

$$\mathcal{I}|\Psi_{\mathrm{CB},I,m_z}\rangle = I|\Psi_{\mathrm{CB},I,m_z}\rangle \quad \mathcal{I}|\Psi_{\mathrm{VB},I,m_z}\rangle = I|\Psi_{\mathrm{VB},I,m_z}\rangle. \tag{27}$$

They are related by chiral symmetry

$$|\Psi_{\mathrm{VB},I,m_z}(\mathbf{M})\rangle = \mathcal{C}|\Psi_{\mathrm{CB},-I,-m_z}(\mathbf{M})\rangle. \tag{28}$$

As $\left[\mathcal{I}, H^{\mathrm{M}}\right] = 0$, the first order perturbation correction from H^{M} is diagonal. For CB1 and CB2 $|\Psi_{\mathrm{CB},I,m_z}(\mathbf{M})\rangle$, we find the perturbation Hamiltonian is

$$\begin{aligned} &\begin{pmatrix} \langle\Psi_{\mathrm{CB},+,m_z}(\mathbf{M})|H^{\mathrm{M}}|\Psi_{\mathrm{CB},+,m_z}(\mathbf{M})\rangle & 0 \\ 0 & \langle\Psi_{\mathrm{CB},-,m_z}(\mathbf{M})|H^{\mathrm{M}}|\Psi_{\mathrm{CB},-,m_z}(\mathbf{M})\rangle \end{pmatrix} \\ &= \begin{pmatrix} \Delta_1\cos\theta_{k_{\mathrm{M}}} & 0 \\ 0 & -\Delta_1\cos\theta_{k_{\mathrm{M}}} \end{pmatrix} \end{aligned} \tag{29}$$

with $\cos\theta_{k_{\mathrm{M}}} = m/\sqrt{m^2 + v^2k_{\mathrm{M}}^2}$, while for VB1 and VB2 $|\Psi_{\mathrm{VB},I,m_z}(\mathbf{M})\rangle$, the perturbation Hamiltonian is

$$\begin{aligned} &\begin{pmatrix} \langle\Psi_{\mathrm{VB},+,m_z}(\mathbf{M})|H^{\mathrm{M}}|\Psi_{\mathrm{VB},+,m_z}(\mathbf{M})\rangle & 0 \\ 0 & \langle\Psi_{\mathrm{VB},-,m_z}(\mathbf{M})|H^{\mathrm{M}}|\Psi_{\mathrm{VB},-,m_z}(\mathbf{M})\rangle \end{pmatrix} \\ &= \begin{pmatrix} -\Delta_1\cos\theta_{k_{\mathrm{M}}} & 0 \\ 0 & \Delta_1\cos\theta_{k_{\mathrm{M}}} \end{pmatrix} \end{aligned}. \tag{30}$$

The eigen-energy of the system at $\mathbf{M}$ after taking into first order perturbation is

$$\begin{aligned} E^{(1)}_{\mathrm{CB},I}(\mathbf{M}) &= E^{\mathrm{TI}}_{\mathrm{CB}}(\mathbf{M}) + \langle\Psi_{\mathrm{CB},I,m_z}(\mathbf{M})|H^{\mathrm{M}}|\Psi_{\mathrm{CB},I,m_z}(\mathbf{M})\rangle \\ E^{(1)}_{\mathrm{VB},I}(\mathbf{M}) &= E^{\mathrm{TI}}_{\mathrm{VB}}(\mathbf{M}) + \langle\Psi_{\mathrm{VB},I,m_z}(\mathbf{M})|H^{\mathrm{M}}|\Psi_{\mathrm{VB},I,m_z}(\mathbf{M})\rangle. \end{aligned} \tag{31}$$

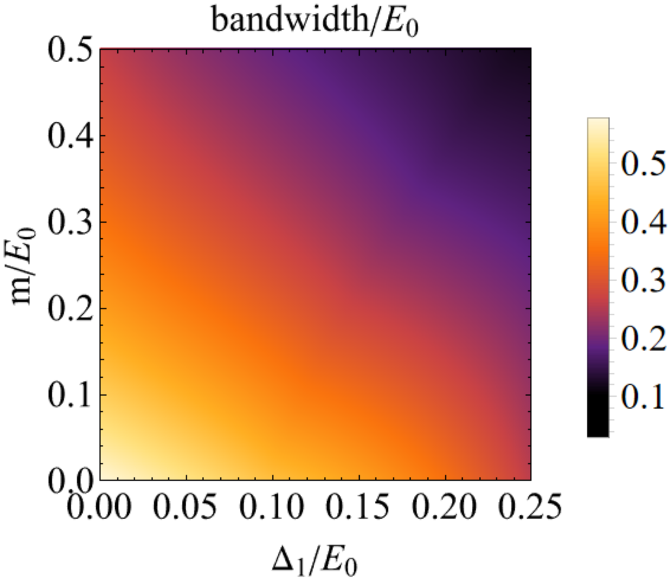

Supplementary Figure 2. Bandwidth of CB1 versus the inter surface hybridization m and moiré potential strength Δ_1. The energy unit $E_0 = v|\mathbf{b}_1^M|$.

The two m_z states are degenerate due to the $\mathcal{TI}$ symmetry so the index m_z is dropped in the above labelling for the eigen-energy. Chiral symmetry leads to

$$\langle\Psi_{\text{VB},I,m_z}(\mathbf{M})|H^{\text{M}}|\Psi_{\text{VB},I,m_z}(\mathbf{M})\rangle = \langle\Psi_{\text{CB},-I,-m_z}(\mathbf{M})|H^{\text{M}}|\Psi_{\text{CB},-I,-m_z}(\mathbf{M})\rangle \tag{32}$$

as $\left[H^{\text{M}}, \mathcal{C}\right] = 0$. If

$$\begin{aligned}\langle\Psi_{\text{CB},+,m_z}(\mathbf{M})|H^{\text{M}}|\Psi_{\text{CB},+,m_z}(\mathbf{M})\rangle > \langle\Psi_{\text{CB},-,m_z}(\mathbf{M})|H^{\text{M}}|\Psi_{\text{CB},-,m_z}(\mathbf{M})\rangle,\\ \langle\Psi_{\text{VB},-,-m_z}(\mathbf{M})|H^{\text{M}}|\Psi_{\text{VB},-,-m_z}(\mathbf{M})\rangle > \langle\Psi_{\text{VB},+,-m_z}(\mathbf{M})|H^{\text{M}}|\Psi_{\text{VB},+,-m_z}(\mathbf{M})\rangle,\end{aligned} \tag{33}$$

which is equivalently

$$\langle\Psi_{\text{VB},-,m_z}(\mathbf{M})|H^{\text{M}}|\Psi_{\text{VB},-,m_z}(\mathbf{M})\rangle > \langle\Psi_{\text{VB},+,m_z}(\mathbf{M})|H^{\text{M}}|\Psi_{\text{VB},+,m_z}(\mathbf{M})\rangle. \tag{34}$$

So, $E^{(1)}_{\text{CB},+}(\mathbf{M}) > E^{(1)}_{\text{CB},-}(\mathbf{M})$ and $E^{(1)}_{\text{VB},-}(\mathbf{M}) > E^{(1)}_{\text{VB},+}(\mathbf{M})$ as shown in Fig. 1(a). CB1 has the eigenstate $|\Psi_{\text{CB},-}(\mathbf{M})\rangle$ with the energy $E^{(1)}_{\text{CB},-}(\mathbf{M})$ while VB1 has the eigenstate $|\Psi_{\text{VB},-}(\mathbf{M})\rangle$ with the energy $E^{(1)}_{\text{VB},-}(\mathbf{M})$. CB1 and VB1 has the same parity at $\mathbf{M}$. The other cases are shown in Fig. 1(b). CB1 and VB1 has the same parity as Eq. (21) for all cases. In the above analysis, the key is that H^{M} commutes with $\mathcal{C}$ and leads to the same parity at $\mathbf{M}$, different from the case at Γ where H^{TI} anti-commutes with $\mathcal{C}$ and results in opposite parities. This leads to one of CB1 and VB1 to be topologically non-trivial while the other to be trivial.

Additionally, from the perturbation perspective, the bandwidth of CB1 can be reduced by increasing m, Δ_1, and moiré lengths as shown in Fig. 2. Increasing m and Δ_1 opens larger gaps at Γ and M and thus flattens CB1. A larger moiré unit cell can reduce $\mathbf{b}_1^M$, as well as E_0, and thus increases both m/E_0 and Δ_1/E_0, leading to the narrowing of the bandwidth of CB1.

B. Topological phase transition when varying ϕ

In this section, we study the topological phase transition of our system when varying ϕ of the moiré potentials. For general ϕ, there is no inversion symmetry. As shown in Fig. 3(a), ν_{CB1} changes from 1 to 0 when ϕ varies from 0 to 1/6. Between the two phases, there is a gap closing around $\phi \approx 1/48$ at K and K'. The gap closing at K, K' happens between two states $|u_{J_z=-1/2}(K)\rangle$ and $|u_{J_z=3/2}(K)\rangle$, belonging to $\bar{K}_6$ and $\bar{K}_4$ irreducible representations as summarized in Tab.I[2, 3], respectively, with different angular momenta $J_z = -1/2$ and $J_z = 3/2$ under three-fold rotation C_3, where $|u_{J_z}(K)\rangle$ are eigen-states of $H_0(K)$ as Eq. (44). The effective Hamiltonian on the basis

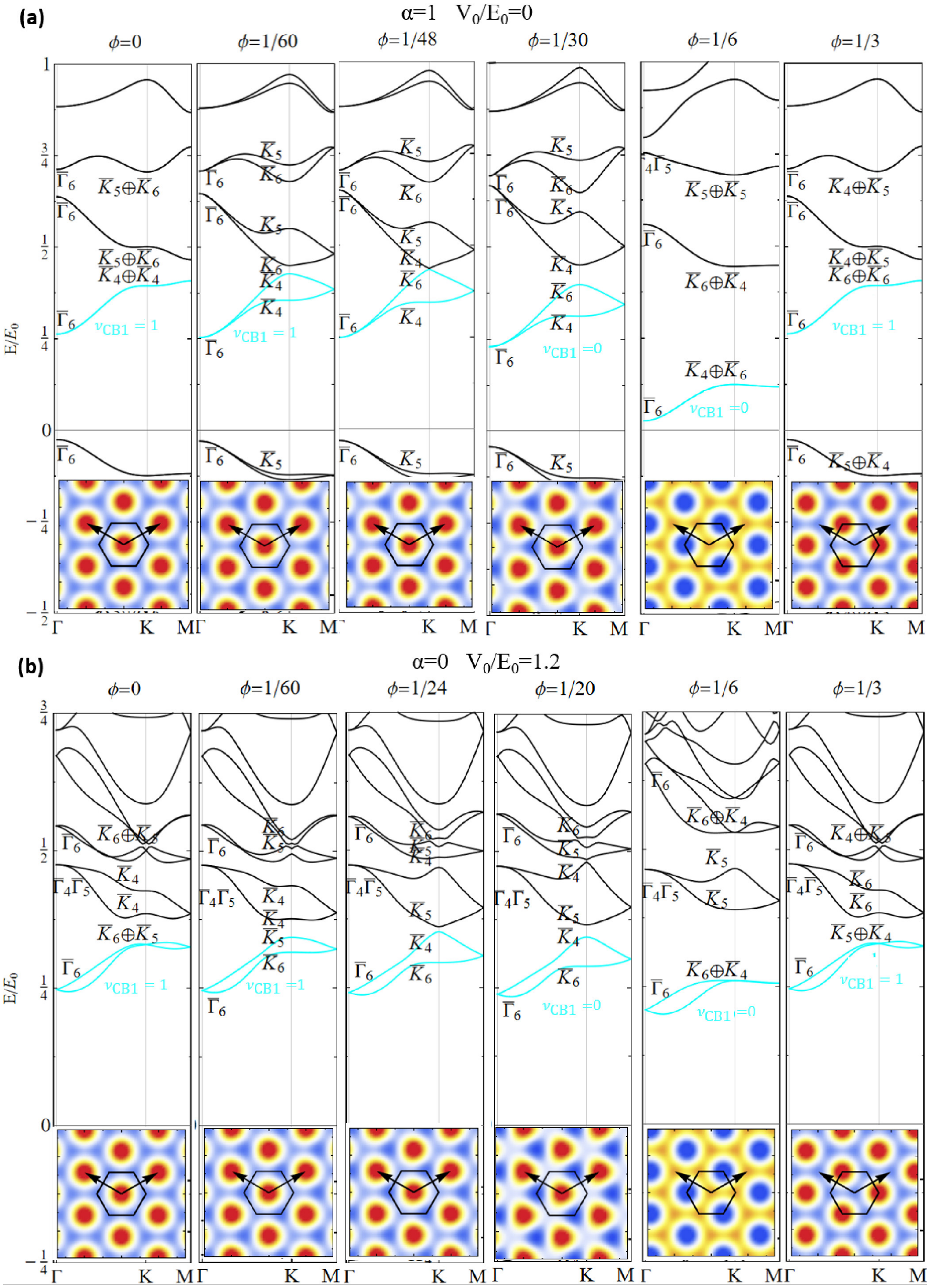

Supplementary Figure 3. (a) Spectra for different ϕ in Fig. 2(a) of the main text. (b) Spectra for different ϕ in Fig. 2(b) of the main text. Insets are real space moiré potentials for different ϕ. States are labelled with the irreducible representations by the little groups C_{3v} at Γ and C_3 at K. $\bar{K}_5$, $\bar{K}_6$, $\bar{K}_4$ represent the angular momentum states under C_3 with $J_z = 1/2, -1/2, 3/2$ respectively. ν is the $\mathbb{Z}_2$ invariant for the lowest conduction bands CB1.

	$\mathcal{C}_3$	$\mathcal{M}_y$	$\mathcal{T}$
$\bar{\Gamma}_4\bar{\Gamma}_5$	$\begin{pmatrix} -1 & 0 \\ 0 & -1 \end{pmatrix}$	$\begin{pmatrix} -i & 0 \\ 0 & i \end{pmatrix}$	$\begin{pmatrix} 0 & -1 \\ 1 & 0 \end{pmatrix}$
$\bar{\Gamma}_6$	$\begin{pmatrix} e^{-i\pi/3} & 0 \\ 0 & e^{i\pi/3} \end{pmatrix}$	$\begin{pmatrix} 0 & -1 \\ 1 & 0 \end{pmatrix}$	$\begin{pmatrix} 0 & 1 \\ -1 & 0 \end{pmatrix}$

(a) Γ

	$\mathcal{C}_3$
$\bar{K}_4$	-1
$\bar{K}_5$	$e^{-i\pi/3}$
$\bar{K}_6$	$e^{i\pi/3}$

(b) K

Supplementary Table I. (a)(b) symmetry operators in the irreducible representation at high symmetry momenta Γ and K for the double space group 156 $P3m1$ corresponding to the point group C_{3v}.

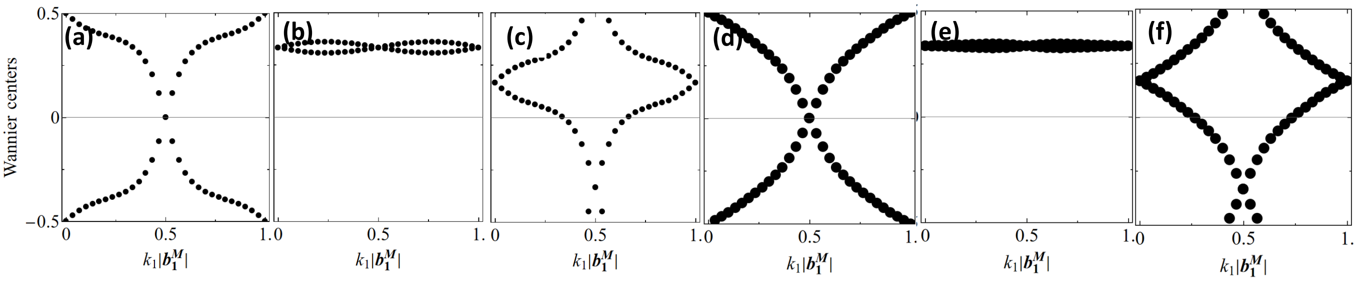

Supplementary Figure 4. (a)(b)(c) The Wannier center flows for CB1 with $\phi = 0, 1/6, 1/3$, corresponding to Fig. 3(a)(c)(d), respectively. (d)(e)(f) The Wannier center flows for CB1 with $\phi = 0, 1/6, 1/3$, corresponding to Fig. 3(e)(g)(h), respectively.

$|u_{J_z=-1/2}(K)\rangle$ and $|u_{J_z=3/2}(K)\rangle$ has the Dirac fermion form $H_{eff}(\mathbf{k}) = v_K(k_x\sigma_x + k_y\sigma_y) + m_K\sigma_z$, up to the linear order, with $\sigma_{x,y,z}$ are Pauli matrices for the two band basis. The gap closing can be captured by one parameter, namely the Dirac mass m_K that is controlled by ϕ, corresponding to the co-dimension 1 case. The $\mathcal{T}$ symmetry guarantees the gap closing also occurring at K', and the gap closings at K and K' lead to the change of $\mathbb{Z}_2$ number ν by 1. The normal insulator (NI) states are localized at moiré potential minima of the Wyckoff position $1b$ shown by the insets of spectrum for $\phi = 1/6$ in Fig. 3(a) (See SM Sec.I.D). From $\phi = 1/6$ to $\phi = 1/3$ in Fig. 3(a), another Dirac-type gap closing should happen at K and K' , and we find the system with $\phi = 1/3$ has $\nu_{\text{CB1}} = 1$.

From the phase diagram in Fig. 2(a)(b) of the main text, we notice that the $\mathbb{Z}_2$ topological property of the system shows a periodicity when ϕ varies by 1/3. Indeed, one can show that the moiré potential $\Delta(r)$ with ϕ and $\Delta'(r)$ with $\phi + 1/3$ (with the same Δ_1 parameter) are related by a constant shift as

$$\begin{aligned}\Delta'(\mathbf{r}) &= \Delta_1 e^{i2\pi\phi} e^{i2\pi/3}\left(e^{i\mathbf{b}_1^{\mathrm{M}}.\mathbf{r}} + e^{i(-\mathbf{b}_1^{\mathrm{M}}+\mathbf{b}_2^{\mathrm{M}}).\mathbf{r}} + e^{i(-\mathbf{b}_2^{\mathrm{M}}).\mathbf{r}}\right) + c.c. \\ &= \Delta_1 e^{i2\pi\phi}\left(e^{i\mathbf{b}_1^{\mathrm{M}}.(\mathbf{r}+\mathbf{a}_1^{\mathrm{M}}/3+2\mathbf{a}_2^{\mathrm{M}}/3)} + e^{i(-\mathbf{b}_1^{\mathrm{M}}+\mathbf{b}_2^{\mathrm{M}}).(\mathbf{r}+\mathbf{a}_1^{\mathrm{M}}/3+2\mathbf{a}_2^{\mathrm{M}}/3)} + e^{i(-\mathbf{b}_2^{\mathrm{M}}).(\mathbf{r}+\mathbf{a}_1^{\mathrm{M}}/3+2\mathbf{a}_2^{\mathrm{M}}/3)}\right) + c.c. \\ &= \Delta(\mathbf{r} + \mathbf{a}_1^{\mathrm{M}}/3 + 2\mathbf{a}_2^{\mathrm{M}}/3).\end{aligned} \tag{35}$$

As a constant shift of potential term cannot change the band topology of the system, ν must keep the same for ϕ and $\phi + 1/3$ while keeping other parameters. For NI phase, the Wyckoff position of Wannier orbitals should also shift accordingly by $\mathbf{a}_1^{\mathrm{M}}/3 + 2\mathbf{a}_2^{\mathrm{M}}/3$, as shown in Fig. 8.

Similar topological phase transitions happen for $\alpha = 0$ and $V_0/E_0 = 1.2$ by a Dirac-type gap closing at K and K' between two states with different angular momenta when ϕ varies from 0 to 1/6 to 1/3, as shown by Fig. 3(b).

The Wannier centers flows for CB1 with $\phi = 0, 1/6, 1/3$ in Fig. 3(a) is shown in Fig. 4(a)-(c). CB1 with $\phi = 0, 1/3$ has nontrivial $\mathbb{Z}_2$ topology as analyzed in the main text. For the case with $\phi = 1/6$, CB1 are topologically trivial. Similarly, the Wannier centers flows for CB1 with $\phi = 0, 1/6, 1/3$ in Fig. 3(b) is shown in Fig. 4(d)-(f). The $\mathbb{Z}_2$ number of CB1 is $\nu_{CB1} = 1$ for $\phi = 0, 1/3$ and $\nu_{CB1} = 0$ for $\phi = 1/6$.

C. Atomic limits at $m_2 \to \infty$

In this section, we will provide theoretical understanding of the non-trivial morié minibands from the atomic limits of the CB1 and CB2 with a large m_2 term (the quadratic term of the inter-surface coupling m), and discuss how the realistic models with a small m_2 are connected to this atomic limit.

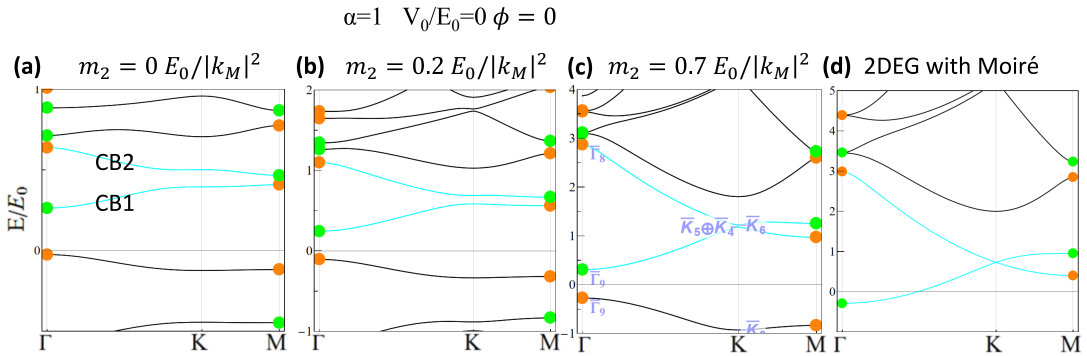

Supplementary Figure 5. (a)(b)(c) Spectra with increasing m_2 for $\alpha = 1, V_0/E_0 = 0, \phi = 0$ of Fig. 2(d) in the main text. Green (Orange) dots denote even (odd) parities at Γ and M. (d) Spectrum of 2DEG on the moiré potential with $\phi = 0$ shown in Fig. 1(c) of the main text. Spectrum in (c) is labelled with irreps by the little group C_{6v} at Γ and C_{3v} at K.

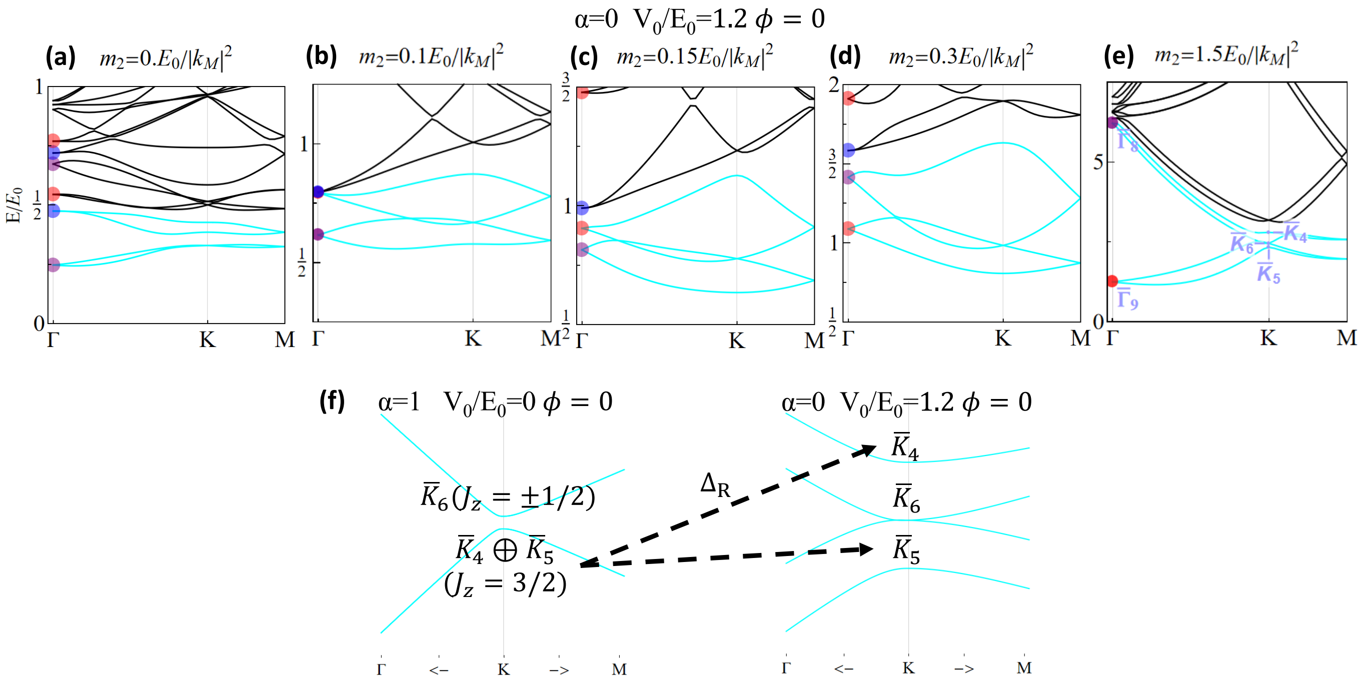

Supplementary Figure 6. (a)-(e) Spectra with increasing m_2 for $\alpha = 0, V_0/E_0 = 1.2, \phi = 0$ of Fig. 2(e) in the main text. Different colorful dots Γ represents different irreps of the little group C_{6v}. Spectrum in (c) is labelled with irreps by the little group C_{6v} at Γ and C_{3v} at K. (f) spectrum around K before and after breaking the inversion symmetry. J_z is the angular momentum of the state at K under C_3.

For $\alpha = 1, V_0/E_0 = 0, \phi = 0$ with the inversion symmetry in Fig. 2(d) of the main text, the energy spectra for increasing m_2 are shown in Fig. 5(a)-(c). We focus on CB1 and CB2 as a whole for atomic limits because they together have $\nu_{CB1} + \nu_{CB2} = 0$ and are topologically trivial. When increasing m_2, we do not find any gap closing between CB1, CB2 and other valence bands or higher conduction bands. Thus, the topological properties of CB1 and CB2 remain the same, and the CB1 and CB2 are adiabatically connected to those corresponding bands in the large m_2 limit. When the m_2 term dominates in H_0, for $\phi = 0$, we may consider the Hamiltonian in the $m_z = \pm i$ basis, Eq. (6), and drop the linear term $\pm iv\hat{k}_\pm$ in the off-diagonal component first. Then, the remaining part of the Hamiltonian just describes the 2D electron gas (2DEG) with a simple parabolic dispersion on a hexagonal potential,

$$H^{\text{2DEG}} = \pm(m_0 - m_2\nabla^2) + \Delta(\mathbf{r}), \tag{36}$$

with $\Delta(\mathbf{r})$ the hexagonal potential, as shown in Fig.1(c) in the main text. The corresponding conduction band dispersion with $m_z = -i$ is shown Fig. 5(d), while the $m_z = +i$ conduction bands are degenerate with $m_z = -i$ bands. The lowest two conduction bands of the Hamiltonian H^{2DEG} can be viewed as coming from two s-wave atomic

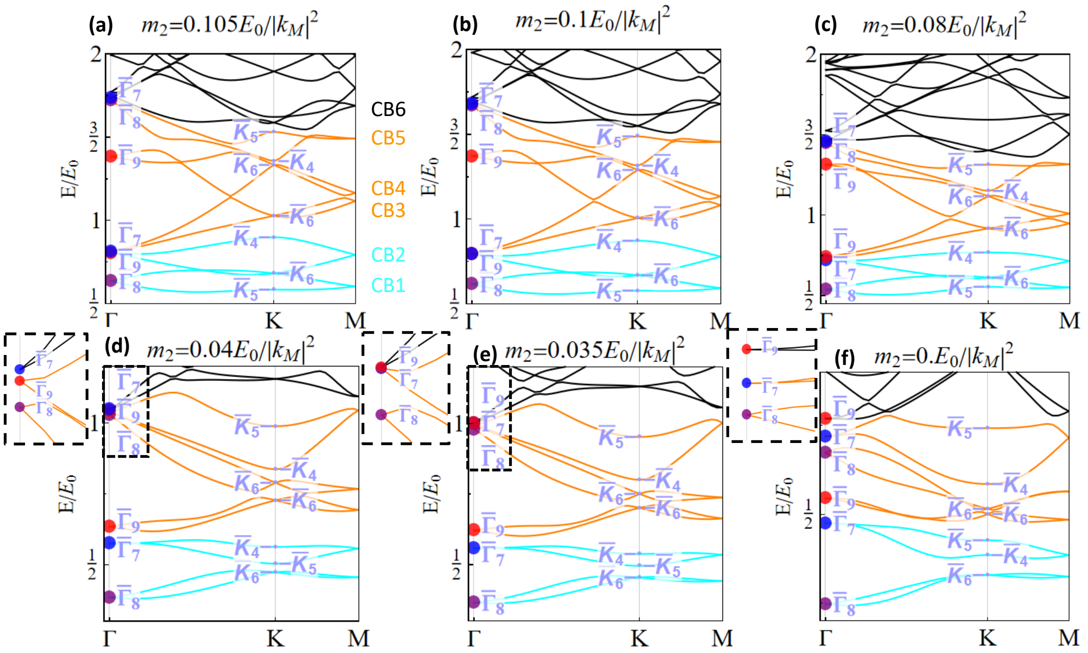

Supplementary Figure 7. (a)-(f) Spectra with reducing m_2 for $\alpha = 0, V_0/E_0 = 1.2, \phi = 0$ of Fig. 2(e) in the main text. Different colorful dots Γ represents different irreps of the little group C_{6v} shown in Tab.II. Cyan bands are CB1 and CB2. Orange ones are Cb3-5. Black ones are CB6 and higher energy bands. Insets in (d)(f) are enlargement of spectra around Γ in the dashed boxes.

	$\mathcal{C}_6$	$\mathcal{M}_y$	$\mathcal{T}$
$\bar{\Gamma}_7$	$\begin{pmatrix} -i & 0 \\ 0 & i \end{pmatrix}$	$\begin{pmatrix} 0 & -1 \\ 1 & 0 \end{pmatrix}$	$\begin{pmatrix} 0 & 1 \\ -1 & 0 \end{pmatrix}$
$\bar{\Gamma}_8$	$\begin{pmatrix} e^{i5\pi/6} & 0 \\ 0 & e^{-i5\pi/6} \end{pmatrix}$	$\begin{pmatrix} 0 & -1 \\ 1 & 0 \end{pmatrix}$	$\begin{pmatrix} 0 & 1 \\ -1 & 0 \end{pmatrix}$
$\bar{\Gamma}_9$	$\begin{pmatrix} e^{i\pi/6} & 0 \\ 0 & e^{-i\pi/6} \end{pmatrix}$	$\begin{pmatrix} 0 & -1 \\ 1 & 0 \end{pmatrix}$	$\begin{pmatrix} 0 & 1 \\ -1 & 0 \end{pmatrix}$

(a) Γ

	$\mathcal{C}_3$	$\mathcal{M}_x$
$\bar{K}_4$	-1	$-i$
$\bar{K}_5$	-1	i
$\bar{K}_6$	$\begin{pmatrix} e^{-i\pi/3} & 0 \\ 0 & e^{i\pi/3} \end{pmatrix}$	$\begin{pmatrix} 0 & -1 \\ 1 & 0 \end{pmatrix}$

(b) K

Supplementary Table II. (a)(b) symmetry operators in the irreducible representation at high symmetry momenta Γ and K for the double space group 183 $P6mm$ corresponding to the point group C_{6v}.

orbitals localized at the moiré hexagonal potential minima of the Wyckoff positions $1b$ and $1c$ for the point group D_{6h} and give rise to a Dirac cone at K and K', similar to the case of graphene. The off-diagonal linear term in Eq. (6) represents the strong spin-orbit-coupling (SOC) of TI thin films, which gives rise to a small gap opening for the dispersion in Fig. 5(c) and can be treated perturbatively. We perform a $\mathbf{k}\cdot\mathbf{p}$ type of perturbation expansion of the full Hamiltonian $H_0(\mathbf{k})$ around K. The basis wave functions are chosen to be the eigen-states of $H_0(\mathbf{K})$ in Eq. 1 of the main eigenstates without SOC ($v_f = 0$)

$$|\tilde{u}_{J_z,m_z}(\mathbf{K})\rangle = |\tilde{u}_{-1/2,-i}(\mathbf{K})\rangle, |\tilde{u}_{3/2,-i}(\mathbf{K})\rangle, |\tilde{u}_{3/2,i}(\mathbf{K})\rangle, |\tilde{u}_{+1/2,i}(\mathbf{K})\rangle \tag{37}$$

for CB1 and CB2 with the irreps $\bar{K}_6$ for $|u_{1/2,+i}(\mathbf{K})\rangle, |u_{-1/2,-i}(\mathbf{K})\rangle$ and $\bar{K}_4$,$\bar{K}_5$ for $|u_{3/2,+i}(\mathbf{K})\rangle, |u_{3/2,-i}(\mathbf{K})\rangle$ (Fig. 5(c)), the detailed forms of which can be numerically evaluated. The relevant symmetry operators are

$$\mathcal{M}_z = -i\sigma_z\tau_0 \quad \mathcal{TI} = i\sigma_y\tau_x\mathcal{K} \tag{38}$$

with σ acts on the different m_z, τ acts on different J_z in one m_z, and $\mathcal{K}$ is the complex conjugate. The SOC couples $|\tilde{u}_{J_z,m_z}(\mathbf{K})\rangle$ and valence bands and contributes a k-independent term from the first order Löwdin perturbation[4] by

$$H_{\rm SOC} = C_0'\sigma_0\tau_0 + \Delta_{\rm KM}\sigma_z\tau_z. \tag{39}$$

The effective Hamiltonian H_{eff} around K to the first order in $\mathbf{k}$ with $m_2 = 0.7E_0/|k_M^2|$ is

$$H_{eff}(\mathbf{k}) \approx H_0(\mathbf{K}) + \left(\frac{\partial H_0(\mathbf{k})}{\partial \mathbf{k}}\right)_{\mathbf{k}=\mathbf{K}} \cdot \mathbf{k} + H_{\rm SOC} = C_0\sigma_0\tau_0 + v_f(k_x\sigma_0\tau_x + k_y\sigma_0\tau_y) + \Delta_{\rm KM}\sigma_z\tau_z, \tag{40}$$

where $C_0, \Delta_{\rm KM}, v_f$ are material dependent parameters and can be obtained numerically from the perturbation expansion. The above effective Hamiltonian $H_{eff}(\mathbf{k})$ resembles the Kane-Mele model [5] with the SOC term $\Delta_{\rm KM}\sigma_z\tau_z$, which provides another understanding of the non-trivial $\mathbb{Z}_2$ topology of the CB1 and CB2 in our moiré system.

For $\alpha = 0, V_0/E_0 = 1.2, \phi = 0$, similar procedure can be applied to find the atomic limits of CB1 and CB2 at a large m_2. The point group in this case is C_{6v} group. For $m_2 = 1.5E_0/|k_M|^2$ in Fig. 6(e), the effective Hamiltonian on the same basis as Eq. (37) is given by

$$H_{eff}(\mathbf{k}) = C_0\sigma_0\tau_0 + v_f(k_x\sigma_0\tau_x + k_y\sigma_0\tau_y) + \Delta_{\rm KM}\sigma_z\tau_z + \Delta_{\rm R}(\sigma_x\tau_y - \sigma_y\tau_x). \tag{41}$$

Besides Kane-Mele SOC term $\Delta_{\rm KM}$, there is another Rashba type of SOC term $\Delta_{\rm R}(\sigma_x\tau_y - \sigma_y\tau_x)$ as the inversion symmetry is broken for $\alpha = 0$ [5]. The Rashba term couples two basis functions $|\tilde{u}_{3/2,\pm i}(\mathbf{K})\rangle$ ($\bar{K}_4$ and $\bar{K}_5$ irreps) and opens the gap between these two states, as schematically shown in Fig. 6(f). The other two states $|\tilde{u}_{1/2,i}(\mathbf{K})\rangle, |\tilde{u}_{1/2,i}(\mathbf{K})\rangle$ ($\bar{K}_6$ irrep) remain degenerate and form a 2D irrep under the little group C_{3v} at K. When this energy splitting $\Delta_{\rm R}$ is larger than the Kane-Mele SOC gap $\Delta_{\rm KM}$, the degenerate states with the 2D irrep $\bar{K}_6$ lies between the $\bar{K}_4$ and $\bar{K}_5$ state, leading to the band touching between CB1 and CB2 bands at K for $m_2 = 1.5E_0/|k_M|^2$ in Fig. 6(e). In this limit, the topology of the CB1 and CB2 is $\nu_{\rm CB1} + \nu_{\rm CB2} = 0$, as the CB1 and CB2 together form an atomic limit. With decreasing m_2 to $m_2 = 0.1E_0/|k_M|^2$, we notice the nodes at K between CB1 and CB2 remains, but there is another band crossing between CB2 and higher conduction bands at Γ in Fig. 6(b). This band crossing at Γ changes the overall $\mathbb{Z}_2$ topology of CB1 and CB2 to $\nu_{\rm CB1} + \nu_{\rm CB2} = 1$ for a smaller m_2. In Fig. 7, we also show the band dispersion and the irreducible representations at high symmetry momenta for other higher-energy minibands (labelled by CB3, CB4, CB5 and CB6). We find the minibands of CB3, CB4 and CB5 are always touching each other and their total $\mathbb{Z}_2$ number is $\nu_{\rm CB3} + \nu_{\rm CB4} + \nu_{\rm CB5} = 1$ for $0.04E_0/|k_M|^2 < m_2 < 0.1E_0/|k_M|^2$. Another transition between CB5 and CB6 occurs at $m_2 \approx 0.035E_0/|k_M|^2$ (See Fig. 7e), and after this transition, $\nu_{\rm CB3} + \nu_{\rm CB4} + \nu_{\rm CB5}$ becomes zero while the other non-trivial $\mathbb{Z}_2$ number is moved to even higher energy minibands. For $m_2 < 0.1E_0/|k_M|^2$, these additional transitions only occur for higher-energy minibands, while the $\mathbb{Z}_2$ topology of CB1 and CB2 remains the same ($\nu_{\rm CB1} + \nu_{\rm CB2} = 1$). For $m_2 < 0.04E_0/|k_M|^2$, we find a gap between CB1 and CB2 opens at K due to the interchange between the $\bar{K}_6$ and $\bar{K}_5$ minibands. Thus, the isolated CB1 with $\nu_{\rm CB1} = 1$ and CB2 with $\nu_{\rm CB2} = 0$ states can be found in Fig. 6(a) for $m_2 = 0$.

D. Normal insulator phases of atomic limits

We construct the maximally localized Wannier functions [6] for the topologically trivial region for the CB1 as shown in Fig. 8. The locations of Wannier functions show the NI phase of CB1 has localized orbitals at Wyckoff positions 1b for $\phi = 1/6$, 1a for $\phi = 1/2$, 1c for $\phi = 5/6$, as indicated in the phase diagram Fig. 2(a)(b) of the main text. Comparing the Wannier functions with the moiré potentials, they are located at minima of moiré potentials and correspond to the lowest conduction bands as expected. Since the minima of potentials change from one to another when tuning ϕ, the localized orbitals shift from one location to the other. The phase transition between two NI phases with orbitals at different Wyckoff positions has gap closing[7], shown as the semi-metal phase in Fig. 2(a)(b) of the main text, as they belong to different atomic limits.

Supplementary Note 2. HARTREE FOCK METHODS FOR COULOMB INTERACTION

A. Eigenbasis projection

In this section, we project the Coulomb interaction into the eigenbasis of the non interacting Hamiltonian $H_0(\mathbf{k})$[8, 9]. The non-interacting moiré Hamiltonian in the second quantization form is

$$H_0(\mathbf{k}) = \sum_{\mathbf{G},\mathbf{G}',\alpha,\alpha'} f^\dagger_\alpha(\mathbf{k}+\mathbf{G})\left(H^{\rm TI}_{\alpha,\alpha'}(\mathbf{k}+\mathbf{G})\delta_{\mathbf{G},\mathbf{G}'} + H^{\rm M}(\mathbf{G}-\mathbf{G}')\delta_{\alpha,\alpha'}\right) f_{\alpha'}(\mathbf{k}+\mathbf{G}'), \tag{42}$$

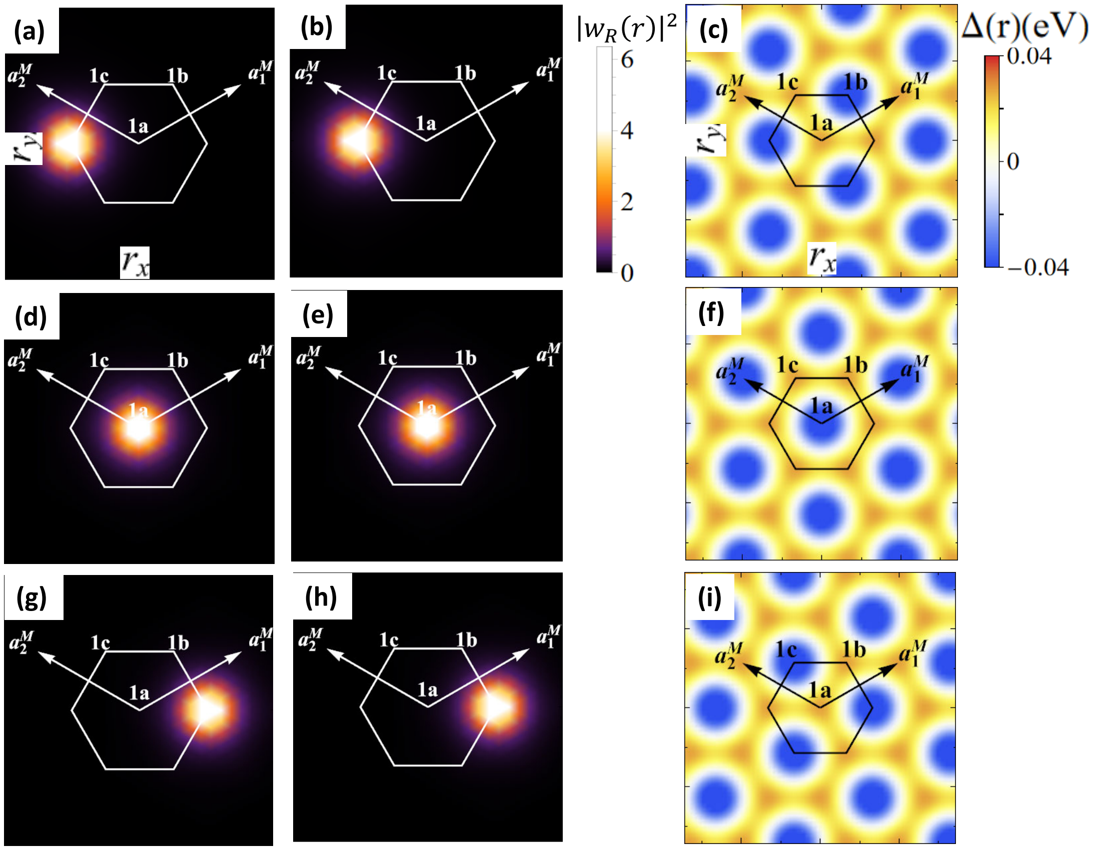

Supplementary Figure 8. (a)(b) The real-space maximally localized Wannier functions $w_{\mathbf{R}}(\mathbf{r})$ for the lowest conduction bands with $\phi = 1/6$ corresponding to Fig. 2(a) of the main text. (c) The real space moiré potentials with $\phi = 1/6$. (d)(e)(f) Those for $\phi = 1/2$ and (g)(h)(i) Those for $\phi = 5/6$.

where $\alpha = 1, ..., 4$ labels both spin and layer index, $f^\dagger_\alpha(\mathbf{k}+\mathbf{G})$ is a fermion creation operator, $\mathbf{k}$ is within the first moiré BZ and $\mathbf{G}$ is Moiré reciprocal lattice vectors. The creation operators for eigenstates of $H_0(\mathbf{k})$ are are defined as

$$c^\dagger_n(\mathbf{k}) = \sum_{\mathbf{G},\alpha} u^n_{\mathbf{G},\alpha}(\mathbf{k}) f^\dagger_\alpha(\mathbf{k}+\mathbf{G}), \tag{43}$$

where $u^n_{\mathbf{G},\alpha}(\mathbf{k})$ satisfies the eigen equation

$$\sum_{\mathbf{G}',\alpha'} \left({H^{\mathrm{TI}}}_{\alpha,\alpha'}(\mathbf{k}+\mathbf{G})\delta_{\mathbf{G},\mathbf{G}'} + H^{\mathrm{M}}(\mathbf{G}-\mathbf{G}')\delta_{\alpha,\alpha'} \right) u^n_{\mathbf{G}',\alpha'}(\mathbf{k}) = E^n_0(\mathbf{k}) u^n_{\mathbf{G},\alpha}(\mathbf{k}) \tag{44}$$

for $H_0(\mathbf{k})$ with energies $E^n_0(\mathbf{k})$. By replacing $\mathbf{G}$ with $\mathbf{G}+\mathbf{G}_0$ and $\mathbf{G}'$ with $\mathbf{G}'+\mathbf{G}_0$ in Eq. (44), we obtain

$$\sum_{\mathbf{G}',\alpha'} \left({H^{\mathrm{TI}}}_{\alpha,\alpha'}(\mathbf{k}+\mathbf{G}_0+\mathbf{G})\delta_{\mathbf{G},\mathbf{G}'} + H^{\mathrm{M}}(\mathbf{G}-\mathbf{G}')\delta_{\alpha,\alpha'} \right) u^n_{\mathbf{G}'+\mathbf{G}_0,\alpha'}(\mathbf{k}) = E^n_0(\mathbf{k}) u^n_{\mathbf{G}+\mathbf{G}_0,\alpha}(\mathbf{k}), \tag{45}$$

which can be viewed as the eigen equations for $u^n_{\mathbf{G},\alpha}(\mathbf{k}+\mathbf{G}_0)$ by replacing $\mathbf{k}$ with $\mathbf{k}+\mathbf{G}_0$ in Eq. (44),

$$\sum_{\mathbf{G}',\alpha'} \left({H^{\mathrm{TI}}}_{\alpha,\alpha'}(\mathbf{k}+\mathbf{G}_0+\mathbf{G})\delta_{\mathbf{G},\mathbf{G}'} + H^{\mathrm{M}}(\mathbf{G}-\mathbf{G}')\delta_{\alpha,\alpha'} \right) u^n_{\mathbf{G}',\alpha'}(\mathbf{k}+\mathbf{G}_0) = E^n_0(\mathbf{k}+\mathbf{G}_0) u^n_{\mathbf{G},\alpha}(\mathbf{k}+\mathbf{G}_0). \tag{46}$$

Thus, we can fix the periodic gauge for the eigen-state as

$$u^n_{\mathbf{G}+\mathbf{G}_0,\alpha}(\mathbf{k}) = u^n_{\mathbf{G},\alpha}(\mathbf{k}+\mathbf{G}_0). \tag{47}$$

As $u^n_{G,\alpha}(\mathbf{k})$ is a set of orthonormal basis, we can take the inverse of the above expansion as

$$f^\dagger_\alpha(\mathbf{k}+\mathbf{G}) = \sum_n u^{n*}_{\mathbf{G},\alpha}(\mathbf{k}) c^\dagger_n(\mathbf{k}) \tag{48}$$

and

$$\left\{c_n(\mathbf{k}), c^\dagger_{n'}(\mathbf{k}')\right\} = \sum_{\mathbf{G},\alpha} u^{n*}_{G,\alpha}(\mathbf{k}) \sum_{\mathbf{G}',\alpha'} u^{n'}_{\mathbf{G}',\alpha'}(\mathbf{k}') \left\{f_\alpha(\mathbf{k}+\mathbf{G}), f^\dagger_{\alpha'}(\mathbf{k}'+\mathbf{G}')\right\} = \delta_{n,n'}\delta(\mathbf{k}-\mathbf{k}'). \tag{49}$$

To improve the efficiency of the numerical calculations, we need to further fix the gauge freedom of eigenstates. An important step is to choose the real gauge for the Hamiltonian and eigenbasis due to the space-time inversion symmetry $C_{2z}\mathcal{T}$ in 2D for moiré potential with $\phi = 0$. Take $C_{2z}\mathcal{T} = U_{\mathrm{CT}}\mathcal{K}$ with $\mathcal{K}$ as complex conjugate. U_{CT} is unitary and satisfies the ${U_{\mathrm{CT}}}^* U_{\mathrm{CT}} = 1$ from $(C_{2z}\mathcal{T})^2 = 1$. Under the basis transformation ${U_{\mathrm{CT}}}^{1/2}$,

$$\left({U_{\mathrm{CT}}}^{1/2}\right)^\dagger C_{2z}\mathcal{T}{U_{\mathrm{CT}}}^{1/2} = {U_{\mathrm{CT}}}^{-1/2} U_{\mathrm{CT}} \left({U_{\mathrm{CT}}}^{1/2}\right)^* \mathcal{K} = \mathcal{K}, \tag{50}$$

and the corresponding Hamiltonian and eigenbasis can be chosen to be real. There is still a $SO(2)$ gauge freedom left for eigenstates for Fig. 2(d) in the main text with inversion and $\pm$ gauge freedom for Fig. 2(e) in the main text without inversion.

In the eigenbasis, the non-interacting Hamiltonian is

$$H_0(\mathbf{k}) = \sum_n c^\dagger_n(\mathbf{k}) E^n_0(k) c_n(\mathbf{k}). \tag{51}$$

The dual-gated Coulomb interaction potential is [8, 10]

$$V(\mathbf{q}) = \frac{e^2 \tanh|\mathbf{q}|d}{2\epsilon_0\epsilon_r|\mathbf{q}|}\frac{1}{S}, \tag{52}$$

where S is the area, d is the dual-gate distance, $\epsilon_0\epsilon_r$ are permittivity, e is electron charge. The Coulomb interaction Hamiltonian in second quantization form is

$$\begin{aligned} H_{\mathrm{I}} =& \frac{1}{2}\sum_{\mathbf{k}_1,\mathbf{k}_2,\mathbf{q},\mathbf{G}} \sum_{\mathbf{G}_1,\alpha_1,\mathbf{G}_2,\alpha_2} V(\mathbf{q}+\mathbf{G}) \\ & f^\dagger_{\alpha_1}(\mathbf{k}_1+\mathbf{G}_1+\mathbf{q}+\mathbf{G}) f^\dagger_{\alpha_2}(\mathbf{k}_2+\mathbf{G}_2-\mathbf{q}-\mathbf{G}) f_{\alpha_2}(\mathbf{k}_2+\mathbf{G}_2) f_{\alpha_1}(\mathbf{k}_1+\mathbf{G}_1) \\ =& \frac{1}{2}\sum_{\mathbf{k}_1,\mathbf{k}_2,\mathbf{q},\mathbf{G}} \sum_{m_1,n_1,m_2,n_2} V(\mathbf{q}+\mathbf{G})\Lambda_{m_1n_1}(\mathbf{k}_1+\mathbf{q}+\mathbf{G},\mathbf{k}_1)\Lambda_{m_2n_2}(\mathbf{k}_2-\mathbf{q}-\mathbf{G},\mathbf{k}_2) \\ & c^\dagger_{m_1}(\mathbf{k}_1+\mathbf{q}) c^\dagger_{m_2}(\mathbf{k}_2-\mathbf{q}) c_{n_2}(\mathbf{k}_2) c_{n_1}(\mathbf{k}_1) \end{aligned} \tag{53}$$

with the form factor

$$\Lambda_{m_1n_1}(\mathbf{k}_1+\mathbf{G},\mathbf{k}_2) = \sum_{\mathbf{G}',\alpha'} u^{m_1*}_{\mathbf{G}',\alpha'}(\mathbf{k}_1+\mathbf{G}) u^{n_1}_{\mathbf{G}',\alpha'}(\mathbf{k}_2) = \langle u^{m_1}(\mathbf{k}_1+\mathbf{G}) | u^{n_1}(\mathbf{k}_2)\rangle. \tag{54}$$

The form factor satisfies

$$\Lambda_{m_1n_1}(\mathbf{k}_1+\mathbf{G},\mathbf{k}_2) = \Lambda^*_{n_1m_1}(\mathbf{k}_2,\mathbf{k}_1+\mathbf{G}) = \Lambda_{m_1n_1}(\mathbf{k}_1,\mathbf{k}_2-\mathbf{G}). \tag{55}$$

In the real eigenbasis, the form factors are all real.

B. Self-consistent Hartree-Fock mean field Theory

In this section, we treat the Coulomb interaction under the Hartree-Fock (HF) approximation[8]. The basic idea is the decoupling of four-fermion operators by

$$\begin{aligned} c^\dagger_1 c_1 c^\dagger_2 c_2 &= \left(\left\langle c^\dagger_1 c_1\right\rangle + c^\dagger_1 c_1 - \left\langle c^\dagger_1 c_1\right\rangle\right)\left(\left\langle c^\dagger_2 c_2\right\rangle + c^\dagger_2 c_2 - \left\langle c^\dagger_2 c_2\right\rangle\right) \\ &\approx \left\langle c^\dagger_1 c_1\right\rangle\left\langle c^\dagger_2 c_2\right\rangle + \left\langle c^\dagger_1 c_1\right\rangle\left(c^\dagger_2 c_2 - \left\langle c^\dagger_2 c_2\right\rangle\right) + \left(c^\dagger_1 c_1 - \left\langle c^\dagger_1 c_1\right\rangle\right)\left\langle c^\dagger_2 c_2\right\rangle \\ &= \left\langle c^\dagger_1 c_1\right\rangle c^\dagger_2 c_2 + c^\dagger_1 c_1\left\langle c^\dagger_2 c_2\right\rangle - \left\langle c^\dagger_1 c_1\right\rangle\left\langle c^\dagger_2 c_2\right\rangle. \end{aligned} \tag{56}$$

The expectation value of the two-fermion operator is the density matrix

$$\rho_{mn}(\mathbf{k}) = \left\langle c_m^\dagger(\mathbf{k}) c_n(\mathbf{k}) \right\rangle = \sum_j \psi_{j,m}^{\mathrm{HF}\,*}(\mathbf{k}) \psi_{j,n}^{\mathrm{HF}}(\mathbf{k}) n_F\left(E_j^{\mathrm{HF}}(\mathbf{k})\right) \tag{57}$$

determined by with n_F as the Fermi distribution function and $\psi_{j,m}^{\mathrm{HF}}(\mathbf{k})$,$E_j^{\mathrm{HF}}(\mathbf{k})$ as the j-th eigenstates and eigen-energies of Hartree-Fock Hamiltonian

$$\sum_m H_{nm}^{\mathrm{HF}}[\rho](\mathbf{k}) \psi_{j,m}^{\mathrm{HF}}(\mathbf{k}) = E_j^{\mathrm{HF}}(\mathbf{k}) \psi_{j,n}^{\mathrm{HF}}(\mathbf{k}), \tag{58}$$

where $H_{nm}^{\mathrm{HF}}[\rho](\mathbf{k})$ is defined in Eq. Eq. (61) below. We always choose $E_{j=1}^{\mathrm{HF}}(\mathbf{k}) < E_{j=2}^{\mathrm{HF}}(\mathbf{k}) < ....$, so the mean field ground state is given by the eigen-state $\psi_{j=1}^{\mathrm{HF}}(\mathbf{k})$. Here, we do not consider non-uniform order parameters in real space with the form $\left\langle c_m^\dagger(\mathbf{k}) c_n(\mathbf{k}+\mathbf{q}) \right\rangle$ for $\mathbf{q} \neq 0$.

The Coulomb interaction under Hartree-Fock approximation is

$$\begin{aligned}
H_{\mathrm{I}}[\rho(\mathbf{k})] = & \frac{1}{2} \sum_{\mathbf{k}_1,\mathbf{k}_2,\mathbf{q},\mathbf{G}} V(\mathbf{q}+\mathbf{G}) \sum_{m_1,n_1,m_2,n_2} \Lambda_{m_1 n_1}\left(\mathbf{k}_1+\mathbf{q}+\mathbf{G}, \mathbf{k}_1\right) \Lambda_{m_2 n_2}\left(\mathbf{k}_2-\mathbf{q}-\mathbf{G}, \mathbf{k}_2\right) \\
& \left(\delta_{\mathbf{q}=0}\left(\rho_{m_1 n_1}\left(\mathbf{k}_1\right) c_{m_2}^\dagger\left(\mathbf{k}_2\right) c_{n_2}\left(\mathbf{k}_2\right) + c_{m_1}^\dagger\left(\mathbf{k}_1\right) c_{n_1}\left(\mathbf{k}_1\right) \rho_{m_2 n_2}\left(\mathbf{k}_2\right) - \rho_{m_1 n_1}\left(\mathbf{k}_1\right) \rho_{m_2 n_2}\left(\mathbf{k}_2\right)\right)\right. \\
& \left. - \delta_{\mathbf{q}=\mathbf{k}_2-\mathbf{k}_1}\left(\rho_{m_1 n_2}\left(\mathbf{k}_2\right) c_{m_2}^\dagger\left(\mathbf{k}_1\right) c_{n_1}\left(\mathbf{k}_1\right) + c_{m_1}^\dagger\left(\mathbf{k}_2\right) c_{n_2}\left(\mathbf{k}_2\right) \rho_{m_2 n_1}\left(\mathbf{k}_1\right) - \rho_{m_1 n_2}\left(\mathbf{k}_2\right) \rho_{m_2 n_1}\left(\mathbf{k}_1\right)\right)\right) \\
= & \sum_{\mathbf{k}_1} C^\dagger\left(\mathbf{k}_1\right)\left(H_{\mathrm{I}}^{\mathrm{H}}[\rho]\left(\mathbf{k}_1\right) - H_{\mathrm{I}}^{\mathrm{F}}[\rho]\left(\mathbf{k}_1\right)\right) C\left(\mathbf{k}_1\right) - E_C[\rho].
\end{aligned} \tag{59}$$

with the Hartree term $H_{\mathrm{I}}^{\mathrm{H}}[\rho]\left(\mathbf{k}_1\right)$, Fock term $H_{\mathrm{I}}^{\mathrm{F}}[\rho]\left(\mathbf{k}_1\right)$, condensation energy $E_C[\rho]$ defined as

$$\begin{aligned}
H_{\mathrm{I}}^{\mathrm{H}}[\rho]\left(\mathbf{k}_1\right) &= \sum_{\mathbf{k}_2,\mathbf{G}} V(\mathbf{G}) \Lambda\left(\mathbf{k}_1-\mathbf{G}, \mathbf{k}_1\right) \operatorname{Tr}\left(\rho\left(\mathbf{k}_2\right) \Lambda^*\left(\mathbf{k}_2-\mathbf{G}, \mathbf{k}_2\right)\right) \\
H_{\mathrm{I}}^{\mathrm{F}}[\rho]\left(\mathbf{k}_1\right) &= \sum_{\mathbf{k}_2,\mathbf{G}} V\left(\mathbf{k}_2-\mathbf{k}_1+\mathbf{G}\right) \Lambda\left(\mathbf{k}_1-\mathbf{G}, \mathbf{k}_2\right) \rho^T\left(\mathbf{k}_2\right) \Lambda^\dagger\left(\mathbf{k}_1-\mathbf{G}, \mathbf{k}_2\right) \\
E_C[\rho] &= \frac{1}{2} \sum_{\mathbf{k}_1,\mathbf{k}_2,\mathbf{G}} V(\mathbf{G}) \operatorname{Tr}\left(\rho\left(\mathbf{k}_1\right) \Lambda^T\left(\mathbf{k}_1-\mathbf{G}, \mathbf{k}_1\right)\right) \operatorname{Tr}\left(\rho\left(\mathbf{k}_2\right) \Lambda^*\left(\mathbf{k}_2-\mathbf{G}, \mathbf{k}_2\right)\right) \\
&\quad - \frac{1}{2} \sum_{\mathbf{k}_1,\mathbf{k}_2,\mathbf{G}} V\left(\mathbf{k}_2-\mathbf{k}_1+\mathbf{G}\right) \operatorname{Tr}\left(\rho^T\left(\mathbf{k}_1\right) \Lambda\left(\mathbf{k}_1-\mathbf{G}, \mathbf{k}_2\right) \rho^T\left(\mathbf{k}_2\right) \Lambda^\dagger\left(\mathbf{k}_1-\mathbf{G}, \mathbf{k}_2\right)\right).
\end{aligned} \tag{60}$$

$C^\dagger(k) = (c_1^\dagger(\mathbf{k}), c_2^\dagger(\mathbf{k})), \ldots, c_n^\dagger(\mathbf{k}))$ with n as the number of bands projected. Since the $H_0(\mathbf{k})$ comes from DFT with Hartree-Fock interaction, the non-interacting states $\psi_{j,m}^{\mathrm{HF}}(k) = \delta_{j,m}$ or $\rho_0(\mathbf{k})$ would be a solution to the Hartree-Fock mean-field Hamiltonian. To achieve this, the $H_{\mathrm{I}}\left[\rho_0\right]$ is subtracted from $H_{\mathrm{I}}\left[\rho\right]$[8, 11]. We define the Hartree-Fock Hamiltonian to be

$$\begin{aligned}
H^{\mathrm{HF}}[\rho](\mathbf{k}) &= H_0(\mathbf{k}) + H_{\mathrm{I}}^{\mathrm{H}}[\rho](\mathbf{k}) - H_{\mathrm{I}}^{\mathrm{F}}[\rho](\mathbf{k}) - \left(H_{\mathrm{I}}^{\mathrm{H}}\left[\rho_0\right](\mathbf{k}) - H_{\mathrm{I}}^{\mathrm{F}}\left[\rho_0\right](\mathbf{k})\right) \\
&= H_0(\mathbf{k}) + H_{\mathrm{I}}^{\mathrm{H}}[\rho-\rho_0](\mathbf{k}) - H_{\mathrm{I}}^{\mathrm{F}}[\rho-\rho_0](\mathbf{k}).
\end{aligned} \tag{61}$$

We solve $H^{\mathrm{HF}}[\rho](\mathbf{k})$ self-consistently in the following standard procedures. We first choose an initial guess of the density matrix, denoted as $\rho_{ini}(\mathbf{k})$, as the order parameter for the filling of one band (half filling in two-band model and one quarter filling for four-band model). Based on $\rho_{ini}(\mathbf{k})$, we can construct $H^{\mathrm{HF}}[\rho_{ini}](\mathbf{k})$ from Eq. (61) and calculate the corresponding new eigenstates that allow us to construct the new density matrix, denoted as $\rho_{new}(\mathbf{k})$. We reset $\rho_{ini}(\mathbf{k}) = \rho_{new}(\mathbf{k})$ and continue the iterative process until the convergence is achieved. The criterion for the convergence is taken as the spectra $\tilde{E}_j^{\mathrm{HF}}(\mathbf{k})$ of $H^{\mathrm{HF}}[\rho_{ini}]$ and $E_j^{\mathrm{HF}}(\mathbf{k})$ of $H^{\mathrm{HF}}[\rho_{new}]$ satisfy

$$\max_{j,\mathbf{k}} |\tilde{E}_j^{\mathrm{HF}}(\mathbf{k}) - E_j^{\mathrm{HF}}(\mathbf{k})| < 10^{-5} E_0 \tag{62}$$

with max taken for all bands j in H^{HF} and $\mathbf{k}$ on the high symmetry lines $\Gamma - K - M$ as shown in Fig. 9(a). $E_0 = v|\mathbf{b}_1^{\mathrm{M}}|$.

The final self-consistent solution for the density matrix is denoted as $\rho^{HF}(\mathbf{k})$ which is determined by the eigen wavefunctions $|\psi_j^{\mathrm{HF}}(\mathbf{k})\rangle$ by Eq. (57) and (58). The energy per particles for each self-consistent solution to the mean-field Hamiltonian is

$$E_{\mathrm{I}}[\rho] = \frac{1}{N} \sum_{\mathbf{k}} \operatorname{Tr} \rho^T(\mathbf{k}) H^{\mathrm{HF}}[\rho](\mathbf{k}) - \left(E_C[\rho] - E_C\left[\rho_0\right]\right). \tag{63}$$

with N as the number of electrons for the filling.

C. Two-band model of CB1

In this section, we discuss the self-consistent solutions of $H^{\rm HF}(\mathbf{k})$ at half filling of two-band model for CB1. Below we will discuss both the inversion-symmetric and asymmetric cases.

We first describe our gauge choice of the non-interacting eigen-states for the case $\alpha = 1, V_0/E_0 = 0, \phi = 0$ with inversion symmetry, which is important to simplify the numerical calculations. The non-interacting states are $|u^{\rm CB1}_{\pm i}(\mathbf{k})\rangle$ with the m_z eigenvalues $\pm i$. The mirror Chern number[12] $C = \pm 1$ can be defined for $|u^{\rm CB1}_{\pm i}(\mathbf{k})\rangle$. The relative phase between two $\mathcal{M}_z$ states are fixed by $C_{2z}\mathcal{T}$ through

$$C_{2z}\mathcal{T}|u^{\rm CB1}_{\pm i}(\mathbf{k})\rangle = |u^{\rm CB1}_{\mp i}(\mathbf{k})\rangle. \tag{64}$$

It turns out that the Hartree-Fock calculations can be simplified by taking the real gauge due to the $C_{2z}\mathcal{T}$ symmetry and thus we transform the basis wavefunctions into the real-gauge form

$$\begin{aligned} |u^{\rm R,CB1}_{+}(\mathbf{k})\rangle &= \frac{1}{\sqrt{2}}\left(e^{{\rm i}\varphi_{\mathbf{k}}}|u^{\rm CB1}_{-i}(\mathbf{k})\rangle + e^{-{\rm i}\varphi_{\mathbf{k}}}|u^{\rm CB1}_{+i}(\mathbf{k})\rangle\right) \\ |u^{\rm R,CB1}_{-}(\mathbf{k})\rangle &= \frac{1}{\sqrt{2}i}\left(e^{{\rm i}\varphi_{\mathbf{k}}}|u^{\rm CB1}_{-i}(\mathbf{k})\rangle - e^{-{\rm i}\varphi_{\mathbf{k}}}|u^{\rm CB1}_{+i}(\mathbf{k})\rangle\right), \end{aligned} \tag{65}$$

where φ_k is the remaining relative $U(1)$ phase between eigen-states opposite m_z (spin $U(1)$ symmetry). The real eigen-states with different φ_k can be related by a $SO(2)$ transformation

$$R(\tilde{\varphi}_{\mathbf{k}}) = \begin{pmatrix} \cos\tilde{\varphi}_{\mathbf{k}} & -\sin\tilde{\varphi}_{\mathbf{k}} \\ \sin\tilde{\varphi}_{\mathbf{k}} & \cos\tilde{\varphi}_{\mathbf{k}} \end{pmatrix}, \tag{66}$$

which shifts $\varphi_{\mathbf{k}}$ to $\varphi_{\mathbf{k}} + \tilde{\varphi}_{\mathbf{k}}$. The other symmetry operators can be taken as

$$\mathcal{T} = i\sigma_y\mathcal{K}; \quad C_{2z} = -i\sigma_y; \quad C_{2z}\mathcal{T} = \mathcal{K}; \quad \mathcal{M}_z = -i\sigma_y, \tag{67}$$

with Pauli matrices σ redefined under the basis $|u^{\rm R,CB1}_{\pm}(\mathbf{k})\rangle$. $\mathcal{M}_z$ is the generator of the $SO(2)$ transformation by $R(\tilde{\varphi}_{\mathbf{k}}) = e^{\tilde{\varphi}_{\mathbf{k}}\mathcal{M}_z}$. $|u^{\rm R,CB1}_{\pm}(\mathbf{k})\rangle$ are taken as the eigenstates projected for the self-consistent Hartree-Fock calculations, which are related to the basis $|u^{\rm CB1}_{m_z=\pm i}\rangle$ used in the main text by Eq. (65). The density matrices in the main text, denoted as $[\rho]_{\alpha\beta} = \langle u^{\rm CB1}_{\alpha}|\hat{\rho}|u^{\rm CB1}_{\beta}\rangle$ with $\alpha, \beta = \pm i$, are related to the density matrices $\rho^{\rm R}$ in the real basis discussed below, denoted as $[\rho^{\rm R}]_{\alpha\beta} = \langle u^{\rm R,CB1}_{\alpha}|\hat{\rho}|u^{\rm R,\ CB1}_{\beta}\rangle$ with $\alpha, \beta = \pm$, by

$$\rho^{\rm R} = U^{\rm R\dagger}\rho U^{\rm R} \tag{68}$$

and

$$U^{\rm R}(\mathbf{k}) = \frac{1}{\sqrt{2}}\begin{pmatrix} e^{i\varphi_{\mathbf{k}}} & -ie^{i\varphi_{\mathbf{k}}} \\ e^{-i\varphi_{\mathbf{k}}} & ie^{-i\varphi_{\mathbf{k}}} \end{pmatrix}, \tag{69}$$

which transforms Pauli matrices σ as $U^{\rm R\dagger}\sigma_y U^{\rm R} = \sigma_x\cos 2\varphi_{\mathbf{k}} - \sigma_z\sin 2\varphi_{\mathbf{k}}, U^{\rm R\dagger}\sigma_x U^{\rm R} = \sigma_x\sin 2\varphi_{\mathbf{k}} + \sigma_z\cos 2\varphi_{\mathbf{k}}, U^{\rm R\dagger}\sigma_z U^{\rm R} = \sigma_y$.

We performed the self-consistent calculations on the basis $|u^{\rm R,CB1}_{\pm}(\mathbf{k})\rangle$ and generally consider the following two types of order parameters: $\rho^{\rm R}_y(\mathbf{k}) = f_{0y}(\mathbf{k})\sigma_0 + f_y(\mathbf{k})\sigma_y$ and $\rho^{\rm R}_{zx}(\mathbf{k}) = f_{0zx}(\mathbf{k})\sigma_0 + f_x(\mathbf{k})\sigma_x + f_z(\mathbf{k})\sigma_z$ with σ acting on the basis $|u^{\rm R,CB1}_{\pm}(\mathbf{k})\rangle$ in Eq. (65). These two types of order parameters possess different symmetry properties as summarized in Tab.III. For $\rho^{\rm R}_y(\mathbf{k})$, the density matrix breaks the $C_{2z}\mathcal{T}$ symmetry with complex $f_y(\mathbf{k})\sigma_y$ and preserves $SO(2)$ symmetry in Eq. (66) by $[R(\tilde{\varphi}_{\mathbf{k}}), \rho^{\rm R}_y(\mathbf{k})] = 0$. $\rho^{\rm R}_y(\mathbf{k})$ also preserves the z-directional mirror symmetry, $[\rho^{\rm R}_y(\mathbf{k}), \mathcal{M}_z] = 0$, as $\mathcal{M}_z = -i\sigma_y$ in the $|u^{\rm R,CB1}_{\pm}(\mathbf{k})\rangle$ basis, which is the generator of the $SO(2)$ symmetry. It represents the many-body states polarized to one of the mirror states $|u^{\rm CB1}_{m_z}\rangle$, dubbed as mirror-polarized states. For $\rho^{\rm R}_{zx}(\mathbf{k})$, the density matrix is real and preserves the $C_{2z}\mathcal{T}$ symmetry ($[C_{2z}\mathcal{T}, \rho^{\rm R}_{zx}(\mathbf{k})] = 0$) but breaks $SO(2)$ symmetry. It represents the many-body states with superposition of both mirror states $|u^{\rm CB1}_{m_z}\rangle$, dubbed as mirror-coherent states. The identity matrix σ_0 appears in both order parameters and mainly determines the filling of states $|\psi^{\rm HF}_j(\mathbf{k})\rangle$ at different momenta $\mathbf{k}$.

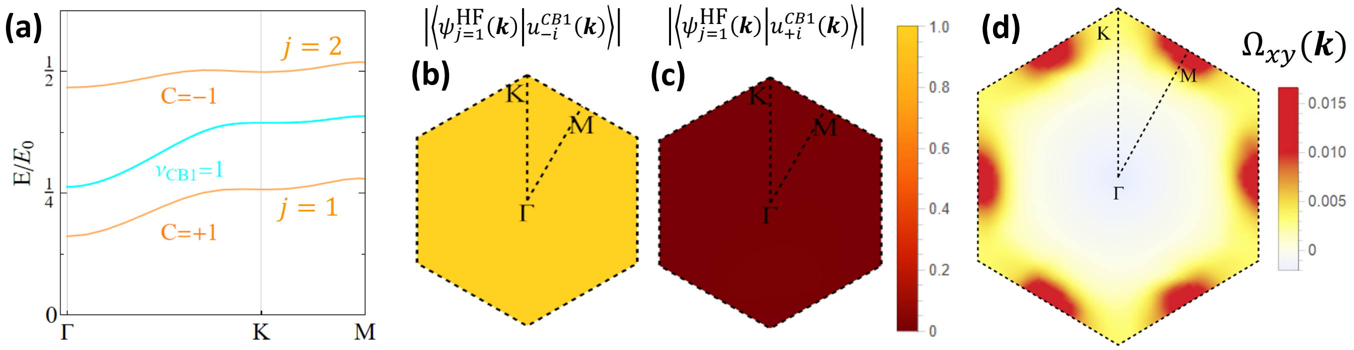

Supplementary Figure 9. (a) The spectrum of $H^{\mathrm{HF}}(\mathbf{k})$ for mirror polarized states with $\rho_y^{\mathrm{R}}(\mathbf{k})$ (orange lines) . (b)(c) The overlap between Hartree-Fock states $|\psi_{j=1}^{\mathrm{HF}}(\mathbf{k})\rangle$ in (a) and the non-interacting mirror polarized basis wavefunction $|u_{\pm i}^{\mathrm{CB1}}\rangle(\mathbf{k})$. (d) The Berry curvature $\Omega_{xy}(\mathbf{k})$ over the moiré BZ for the filled band. Here the calculation is for the two-band model with the parameters $\phi=0,\alpha=1,V_0/E_0=0$.

	$\mathcal{T}$	C_{2z}	$C_{2z}\mathcal{T}$	$\mathcal{M}_z$	$\mathcal{I}$	C_{6z}	C_{3z}	$\mathcal{M}_x$	$\mathcal{M}_y$
	$i\tau_0 s_y\mathcal{K}$	$-i\tau_0 s_z$	$i\tau_0 s_x\mathcal{K}$	$-i\tau_x s_z$	$\tau_x s_0$	$\exp(-i\pi\tau_0 s_z/6)$	$\exp(-i\pi\tau_0 s_z/3)$	$-i\tau_0 s_x$	$-i\tau_0 s_y$
	$i\sigma_y\mathcal{K}$	$-i\sigma_y$	$\mathcal{K}$	$-i\sigma_y$	σ_0	$\exp(-i\pi\sigma_y/6)$	$\exp(-i\pi\sigma_y/3)$	$-i\sigma_z$	$-i\sigma_x$
$\rho_y^{\mathrm{R,HF}}(\mathbf{k})$	×	✓	×	✓	✓	✓	✓	×	×
$\rho_{zx}^{\mathrm{R,HF}}(\mathbf{k})$	×	×	✓	×	✓	×	×	✓	×

Supplementary Table III. A summary of symmetries preserved (✓) or broken (×) by the mirror polarized states with $\rho_y^{\mathrm{R,HF}}(\mathbf{k})$ and the mirror coherent states with $\rho_{zx}^{\mathrm{R,HF}}(\mathbf{k})$. $\rho^{\mathrm{R,HF}}(\mathbf{k})$ are the self-consistent solutions from the mean-field Hamiltonian $H^{\mathrm{HF}}(\mathbf{k})$. The symmetry operators are written in two basis. τ, s are Pauli matrices for the surface and spin basis as Eq.1 in the main text. σ are the Pauli matrices for the real basis $|u_{\pm}^{\mathrm{R,CB1}}(\mathbf{k})\rangle$.

Different symmetry properties of $\rho_y^{\mathrm{R}}(\mathbf{k})$ and $\rho_{zx}^{\mathrm{R}}(\mathbf{k})$ under the $C_{2z}\mathcal{T}$ and $SO(2)$ symmetry guarantee that they will not mix with each other. We may start from the initial density matrix $\rho_{ini}^{\mathrm{R}}(\mathbf{k})=\rho_y^{\mathrm{R}}(\mathbf{k})$ with certain forms of f_{0y} and f_y, which preserves the $R(\tilde{\varphi}_{\mathbf{k}})$ symmetry, $[\rho_{ini}^{\mathrm{R}}(\mathbf{k}),R(\tilde{\varphi}_{\mathbf{k}})]=0$. As the Hartree-Fock Hamiltonian $H^{\mathrm{HF}}[\rho_{ini}^{\mathrm{R}}](\mathbf{k})$ is constructed from ρ_{ini}^{R}, direct calculation shows that $[H^{\mathrm{HF}}[\rho_{ini}^{\mathrm{R}}](\mathbf{k}),R(\tilde{\varphi}_{\mathbf{k}})]=0$ for any $\varphi_{\mathbf{k}}$. From Eq. (66) of $R(\tilde{\varphi}_{\mathbf{k}})$, the Hamiltonian has to take the form

$$H^{\mathrm{HF}}[\rho_{ini}^{\mathrm{R}}](\mathbf{k})=h_0(\mathbf{k})\sigma_0+h_y(\mathbf{k})\sigma_y, \tag{70}$$

where $h_0(\mathbf{k}),h_y(\mathbf{k})$ are some functions of $\mathbf{k}$ which can be determined numerically. From the above form of the Hamiltonian, the new density matrix can be evaluated as

$$\rho_{new}^{\mathrm{R}}(\mathbf{k})=\sum_{j=\pm}n_F\left(h_0(\mathbf{k})+jh_y(\mathbf{k})\right)\frac{1}{2}\left(\sigma_0-j\sigma_y\right), \tag{71}$$

which still satisfies $[\rho_{new}^{\mathrm{R}}(\mathbf{k}),R(\tilde{\varphi}_{\mathbf{k}})]=0$. $n_F(E)$ is the Fermi distribution function. Thus, the $R(\tilde{\varphi}_{\mathbf{k}})$ symmetry is preserved in the self-consistent calculation process and thus the Pauli matrices σ_x and σ_z cannot be generated in the final $\rho_y^{\mathrm{R,HF}}(\mathbf{k})$.

Similar argument can be applied to the initial density matrix $\rho_{ini}^{\mathrm{R}}(\mathbf{k})=\rho_{zx}^{\mathrm{R}}(\mathbf{k})$ with certain forms of f_{0zx},f_z,f_x. The $C_{2z}\mathcal{T}$ symmetry is preserved for $\rho_{ini}^{\mathrm{R}}(\mathbf{k})$ and $H^{\mathrm{HF}}[\rho_{ini}^{\mathrm{R}}](\mathbf{k})$. As a result, the Hamiltonian form has to be

$$H^{\mathrm{HF}}=h_0(\mathbf{k})\sigma_0+h_x(\mathbf{k})\sigma_x+h_z(\mathbf{k})\sigma_z, \tag{72}$$

and the new density matrix is

$$\rho_{new}^{\mathrm{R}}(\mathbf{k})=\sum_{j=\pm}n_F\left(h_0(\mathbf{k})+j\sqrt{h_x^2(\mathbf{k})+h_z^2(\mathbf{k})}\right)\frac{1}{2}\left(\sigma_0+j\frac{h_x(\mathbf{k})}{\sqrt{h_x^2(\mathbf{k})+h_z^2(\mathbf{k})}}\sigma_x+j\frac{h_z(\mathbf{k})}{\sqrt{h_x^2(\mathbf{k})+h_z^2(\mathbf{k})}}\sigma_z\right), \tag{73}$$

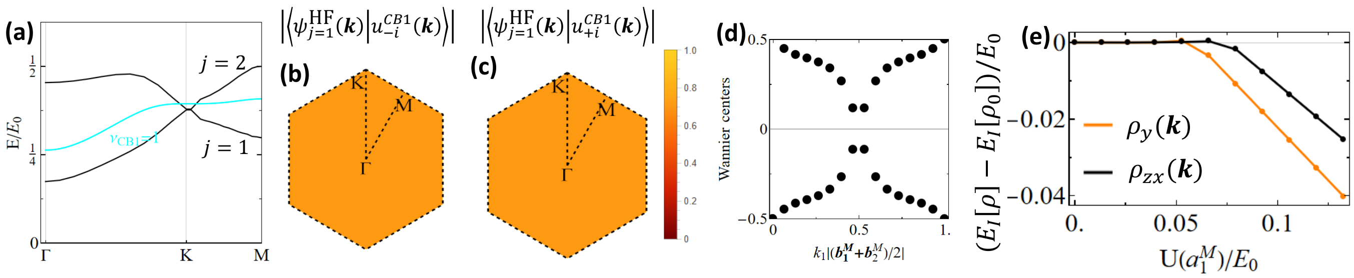

Supplementary Figure 10. (a) The spectrum of $H^{\mathrm{HF}}(\mathbf{k})$ for the mirror coherent states with $\rho^{\mathrm{R}}_{zx}(\mathbf{k})$ (black lines) . (b)(c) The overlap between mirror coherent states $|\psi^{HF}_{j=1}(\mathbf{k})\rangle$ for the filled band in (a) and the non interacting states $|u^{\mathrm{CB1}}_{\pm i}\rangle(\mathbf{k})$. (d) The Wannier center flow for both eigen-states of $H^{\mathrm{HF}}(\mathbf{k})$. (e) The energy per particle $E_{\mathrm{I}}[\rho^{\mathrm{R}}]$ with non-interacting energy $E_{\mathrm{I}}[\rho_0]$ subtracted for $\rho^{\mathrm{R}}_y(\mathbf{k})$ and $\rho^{\mathrm{R}}_{zx}(\mathbf{k})$. Here the calculation is for the two-band model with the parameters $\phi=0,\alpha=1,V_0/E_0=0$.

which has the $C_{2z}\mathcal{T}$ symmetry, $[\rho^{\mathrm{R}}_{new}(\mathbf{k}),C_{2z}\mathcal{T}]=0$. So the Pauli matrix σ_y cannot be mixed into the density matrix $\rho^{\mathrm{R,HF}}_{zx}(\mathbf{k})$ in the above procedure. Based on this symmetry argument, we can discuss the self-consistent solutions for the density matrix form $\rho^{\mathrm{R}}_y(\mathbf{k})$ and $\rho^{\mathrm{R}}_{zx}(\mathbf{k})$, separately, below.

For $\rho^{\mathrm{R}}_y(\mathbf{k})$, we choose the initial density matrix as

$$\rho^{\mathrm{R}}_{ini}(\mathbf{k})=\frac{1}{2}(\sigma_0-\sigma_y), \tag{74}$$

which can be obtained from the states $|u^{\mathrm{CB1}}_{-i}(\mathbf{k})\rangle$. Although the initial density matrix ρ^{R}_{ini} is independent of $\mathbf{k}$, the $H^{\mathrm{HF}}(\mathbf{k})$ in Eq. (61) depends on $\mathbf{k}$ and the self-consistent density matrix should in principle depend on $\mathbf{k}$. The self-consistent solutions are shown in Fig. 9, in which we evaluate the overlap

$$|\langle\psi^{\mathrm{HF}}_{j=1}(\mathbf{k})|u^{\mathrm{CB1}}_{-i}(\mathbf{k})\rangle|=1 \quad |\langle\psi^{\mathrm{HF}}_{j=1}(\mathbf{k})|u^{\mathrm{CB1}}_{+i}(\mathbf{k})\rangle|=0 \tag{75}$$

in Fig. 9(b)(c) with $|\psi^{\mathrm{HF}}_{j=1}(\mathbf{k})\rangle=\sum_{m=\pm}\psi^{\mathrm{HF}}_{1,m}(\mathbf{k})|u^{\mathrm{R},CB1}_m(\mathbf{k})\rangle$ in Fig. 9(a) for the filled bands at half-filling. Furthermore, the Chern number for the band j can be evaluated by

$$C=\frac{1}{2\pi}\int d^2\mathbf{k}\;\Omega_{xy}(\mathbf{k}), \tag{76}$$

where the Berry curvature is calculated by [13]

$$\begin{aligned}\Omega_{xy}(\mathbf{k})=-\arg\Big(&\langle\psi^{\mathrm{HF}}_j(\mathbf{k})|\psi^{\mathrm{HF}}_j(\mathbf{k}+\delta k_x)\rangle\langle\psi^{\mathrm{HF}}_j(\mathbf{k}+\delta k_x)|\psi^{\mathrm{HF}}_j(\mathbf{k}+\delta k_x+\delta k_y)\rangle\\ &\langle\psi^{\mathrm{HF}}_j(\mathbf{k}+\delta k_y)|\psi^{\mathrm{HF}}_j(\mathbf{k}+\delta k_x+\delta k_y)\rangle^{-1}\langle\psi^{\mathrm{HF}}_j(\mathbf{k})|\psi^{\mathrm{HF}}_j(\mathbf{k}+\delta k_y)\rangle^{-1}\Big)\end{aligned} \tag{77}$$

with $\delta k_x,\delta k_y$ as the momenta connecting neighboring momentum grid points in the x,y direction. Our calculation shows $C=+1$ for the filled band $|\psi^{\mathrm{HF}}_{j=1}(\mathbf{k})\rangle$ with the Berry curvature distribution shown in Fig. 9(d).

For $\rho^{\mathrm{R}}_{zx}(\mathbf{k})$, the initial density matrices are taken as

$$\rho^{\mathrm{R}}_{ini}=\frac{1}{2}(\sigma_0+\sigma_z\cos 2\tilde{\varphi}-\sigma_x\sin 2\tilde{\varphi}) \tag{78}$$

for a certain uniform value of $\tilde{\varphi}$, which corresponds to states $\cos\tilde{\varphi}|u^{\mathrm{R,CB1}}_{+}(\mathbf{k})\rangle-\sin\tilde{\varphi}|u^{\mathrm{R,CB1}}_{-}(\mathbf{k})\rangle$. The HF energy spectrum from this initial ρ^{R}_{ini} in Fig. 10 shows nodes at K,K'. These nodes can be understood from nonzero Euler number, denoted as μ, a topological invariant defined for a two-band model with the $C_{2z}\mathcal{T}$ symmetry [14–16]. The non-interacting eigen-state of CB1 has non-trivial $\mathbb{Z}_2$ number $\nu_{\mathrm{CB1}}=1$, and the Euler number can be related to the $\mathbb{Z}_2$ number by $\nu_{CB1}=\mu \mod 2$[16]. Thus, when $\nu_{\mathrm{CB1}}=1$, μ has to be an odd number, which gives rise to 2μ of gapless Dirac nodes in the spectrum. Because $C_{2z}\mathcal{T}$ is preserved for the initial density matrix ρ^{R}_{ini}, this symmetry remains throughout the whole self-consistent calculation process, so Euler class is still well-defined for the final self-consistent Hatree-Fock ground state. We evaluate the Wannier center flow for the final Hatree-Fock ground state, which is shown in Fig. 10(d). The nonzero Euler class with $\mu=1$ from the Wannier center flow guarantees the existence of 2

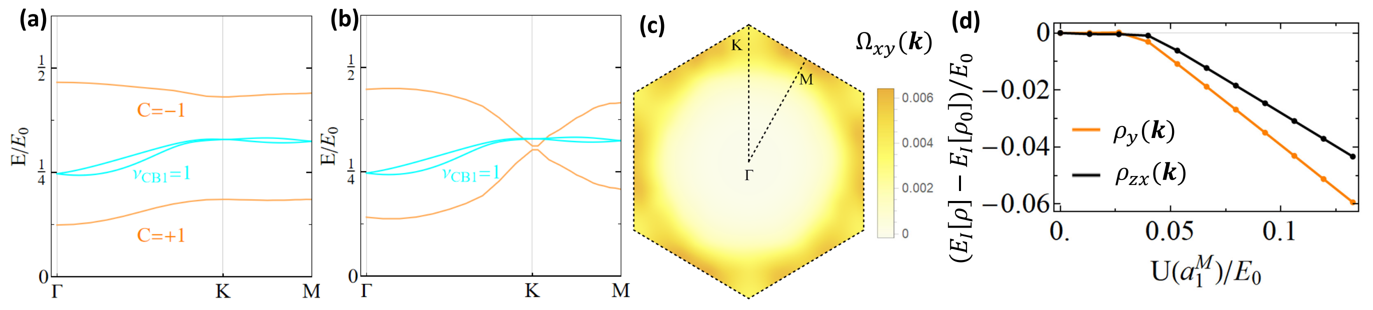

Supplementary Figure 11. (a)(b) The spectra (orange) of $H^{\rm HF}(\mathbf{k})$ with $\rho_y^{\rm R}(\mathbf{k})$ for (a) and $\rho_{zx}^{\rm R}(\mathbf{k})$ for (b). The cyan lines are non-interacting spectrum. (c) Berry curvature for the lower band of $H^{\rm HF}(\mathbf{k})$ in (a). (c) The energy per particle $E_{\rm I}[\rho^{\rm R}]$ with non-interacting energy $E_{\rm I}[\rho_0]$ subtracted for $\rho_y^{\rm R}(\mathbf{k})$ and $\rho_{zx}^{\rm R}(\mathbf{k})$. Here the calculation is for the two-band model with the parameters $\phi = 0, \alpha = 0, V_0/E_0 = 1.2$.

Dirac nodes in the Hartree-Fock spectrum. Fig. 10(b)(c) shows that the Hartree-Fock solutions $|\psi^{\rm HF}_{j=1}(\mathbf{k})\rangle$ shown in Fig. 10(a) are superposition of two m_z states with the same probability

$$|\langle\psi^{\rm HF}_{j=1}(\mathbf{k})|u^{\rm CB1}_{-i}(\mathbf{k})\rangle| = 1/\sqrt{2} \quad |\langle\psi^{\rm HF}_{j=1}(\mathbf{k})|u^{\rm CB1}_{+i}(\mathbf{k})\rangle| = 1/\sqrt{2}, \tag{79}$$

which are denoted as mirror coherent states.

The true ground state of the system is obtained by comparing the energies $E_{\rm I}[\rho^{\rm R}]$ of two self-consistent density matrices in Fig. 10(e). Above the critical interaction value around $0.05E_0 \approx 2$ meV, our calculation shows that the mirror polarized state with $\rho_y^{\rm R}(\mathbf{k})$ has lower energies than the non-interacting ground state and the mirror coherent states with $\rho_{zx}^{\rm R}(\mathbf{k})$. This is because non-interacting ground state and mirror coherent state have gapless excitations in their spectrum, while the mirror polarized states are fully gapped. Thus, we conclude that the true ground state is a mirror polarized Chern insulator.

For the case with $\alpha = 0, V_0/E_0 = 1.2, \phi = 0$ without inversion, the mirror symmetry $\mathcal{M}_z$ is broken so we cannot characterize the non-interacting eigen-state with mirror eigen-values and mirror Chern number. However, the $C_{2z}\mathcal{T}$ symmetry remains, so we can still choose the real gauge for non-interacting states as $|u_1^{\rm R,CB1}(\mathbf{k})\rangle, |u_2^{\rm R,CB1}(\mathbf{k})\rangle$, which satisfies

$$C_{2z}\mathcal{T}|u_n^{\rm R,CB1}(\mathbf{k})\rangle = |u_n^{\rm R,CB1}(\mathbf{k})\rangle, \tag{80}$$

where $n = 1, 2$ labels two spin-split bands for the Kramers' pair of CB1. Consequently, two types of order parameters, $\rho_y^{\rm R}(\mathbf{k})$ that breaks $C_{2z}\mathcal{T}$ and $\rho_{zx}^{\rm R}(\mathbf{k})$ that breaks spin $U(1)$ symmetry, do not mix with each other. The self-consistent solutions with two types of order parameters are shown in Fig. 11. $\rho_y^{\rm R}(\mathbf{k})$ breaks $\mathcal{T}$ symmetry and one band of CB1 with nonzero Chern number is gapped from the other band. $\rho_{zx}^{\rm R}(\mathbf{k})$ has Dirac nodes in spectra at K, K' with energies $E_{\rm I}[\rho_{zx}^{\rm R}(\mathbf{k})]$ higher than the other case. The ground state is an interaction-driven Chern insulator, same as the inversion symmetric case.

D. Four-band model with CB1 and CB2

In the main text, we have discussed the important role of the band mixing between CB1 and CB2 induced by the Coulomb interaction, which can result in the interacting ground state varying from the QAH state to a trivial insulator state for the realistic Coulomb interaction strength for the inversion symmetric case ($V_0 = 0$), while the QAH state remains for the realistic Coulomb interaction when a large asymmetric potential V_0 is applied. The difference between the inversion symmetric and asymmetric cases is that both CB1 and CB2 carry non-trivial $\mathbb{Z}_2$ number, $\nu_{CB1} = \nu_{CB2} = 1$, for inversion symmetric case, while a strong asymmetric potential V_0 gives a trivial insulator phase for CB2, $\nu_{CB1} = 1$ and $\nu_{CB2} = 0$, for inversion asymmetric case. This effect can only be taken into account when considering both CB1 and CB2, and thus it is important to go beyond the two-band model discussed above and consider a four-band model with both CB1 and CB2. In this section, we will provide more details of our numerical self-consistent calculations of the interacting ground state within the HF approximations for the four-band model. Below we always assume the 1/4 filling of four bands, which corresponds to the 1/2 filling of CB1.

For the case $\alpha = 1, V_0/E_0 = 0, \phi = 0$ with inversion symmetry, the non-interacting states now have $|u^{\rm CB1}_{\pm i}(\mathbf{k})\rangle$ with $C = \pm 1$ and $|u^{\rm CB2}_{\pm i}(\mathbf{k})\rangle$ with $C = \mp 1$, where C denotes the Chern number of the minibands in the $m_z = -i$

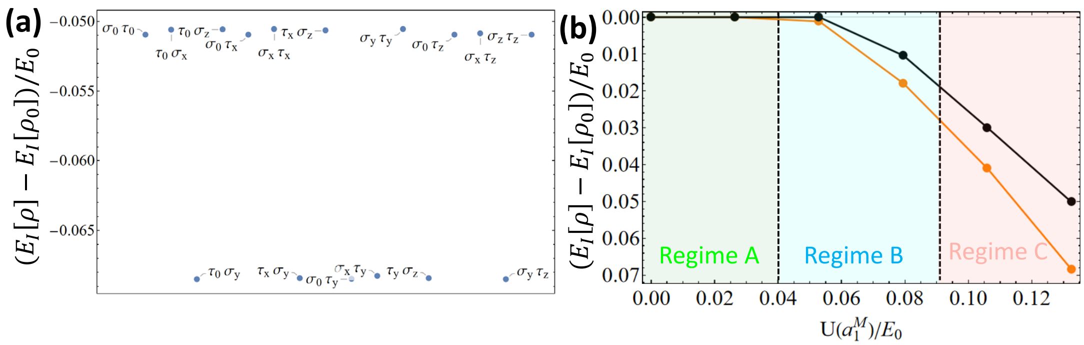

Supplementary Figure 12. (a) The energy per particle for the self-consistent Hartree-Fock solutions labelled by initial density matrix ρ_{ini} for $U(a_1^{\mathrm{M}}) = 0.13E_0$. (b) The energy per particle for the self-consistent Hartree-Fock solutions under different interaction strength. Orange (Black) lines are $C_{2z}\mathcal{T}$ symmetry breaking (preserving) states. Here the calculation is for the four-band model with the parameters $\phi = 0, \alpha = 1, V_0/E_0 = 0$

.

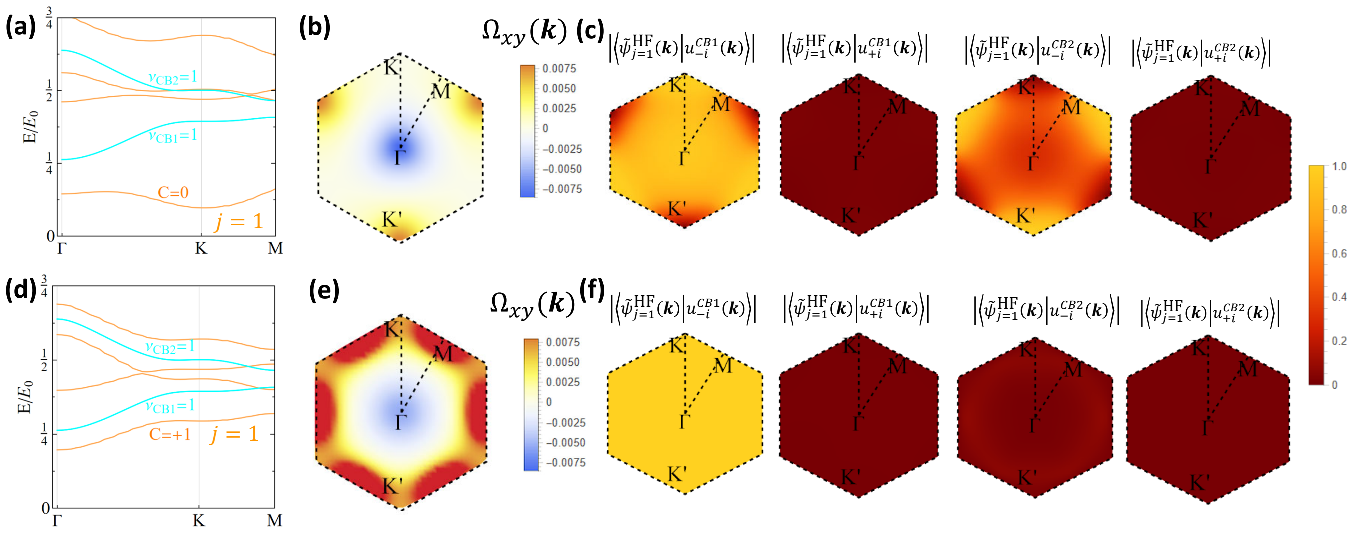

Supplementary Figure 13. (a) The spectra (orange) of $H^{\mathrm{HF}}(\mathbf{k})$ with $\rho^{\mathrm{R}}_{ini}(\mathbf{k}) = \tau_0\sigma_y$ and $U(a_1^{\mathrm{M}}) = 0.13E_0$. The cyan lines are non-interacting spectrum. (b) Berry curvature for the lowest band $j = 1$ of $H^{\mathrm{HF}}(\mathbf{k})$ in (a). (c) The overlap between the ground states $|\tilde{\psi}^{\mathrm{HF}}_{j=1}(\mathbf{k})\rangle$ in (a) and the non-interacting states $|u^{\mathrm{R,CB1/CB2}}_{\pm i}(\mathbf{k})\rangle$. (d)(e)(f) are same as (a)(b)(c), respectively, for $U(a_1^{\mathrm{M}}) = 0.08E_0$. Here the calculation is for the four-band model with the parameters $\phi = 0, \alpha = 1, V_0/E_0 = 0$.

subspace (mirror Chern number). For the convenience of the calculations, we choose the real gauge by applying the transformation given in Eq. (65) to the basis wave-functions for both CB1 and CB2, denoted as $|u^{\mathrm{R,CB1}}_{\pm}\rangle, |u^{\mathrm{R,CB2}}_{\pm}\rangle$. The initial density matrices ρ^{R}_{ini} are taken as one of $\tau_i\sigma_j$ with $i, j = 0, x, y, z$ and τ acting on CB1,CB2 and σ acts on two real basis in one Kramer pair of bands, which are all possible 4×4 uniform density matrices. From $E_I[\rho^{\mathrm{R}}(\mathbf{k})]$ for different ρ^{R}_{ini} in Fig. 12, the self-consistent solutions can also be divided into two groups: one with complex density matrices breaking $C_{2z}\mathcal{T}$ (e.g. $\tau_i\sigma_y$ and $\tau_y\sigma_i$ with $i = 0, x, z$) and the other with real density matrices preserving breaking $C_{2z}\mathcal{T}$ (e.g. $\tau_i\sigma_j$ with $i, j = 0, x, z$ and $\tau_y\sigma_y$), as the $C_{2z}\mathcal{T}$ symmetry is preserved at the single-particle Hamiltonian level for $\phi = 0$. We generally find that the self-consistent solutions with the initial complex density matrices have lower energies, as shown in Fig. 12(a). Although the initial density matrices ρ^{R}_{ini} are different, we numerically find the self-consistent density matrices are all mirror polarized states related by C_{2z} or $\mathcal{T}$. From Fig.4(a) in the main text or Fig. 12(b) reproduced here, the self-consistent solutions with complex density matrices becomes the ground states when the Coulomb interaction exceeds $0.04E_0$. The inter-band mixing between CB1 and CB2 is negligible for interaction strength in regime B with $0.04E_0 < U(a_1^M) < 0.09E_0$ but a strong band mixing is found in regime C with larger Coulomb interaction $0.09E_0 < U(a_1^M)$. Fig. 13 shows the self-consistent solutions for the initial density matrix $\rho^{\mathrm{R}}_{ini} = \tau_0\sigma_y$ as an example. Here Fig. 13(a)-(c) are for $U(a_1^{\mathrm{M}}) = 0.13E_0$ (regime C) while (d)-(f) are for $U(a_1^{\mathrm{M}}) = 0.08E_0$ (regime B). The filled band $|\tilde{\psi}^{\mathrm{HF}}_{j=1}(\mathbf{k})\rangle$ in Fig. 13(a) for regime C has the Chern number $C = 0$

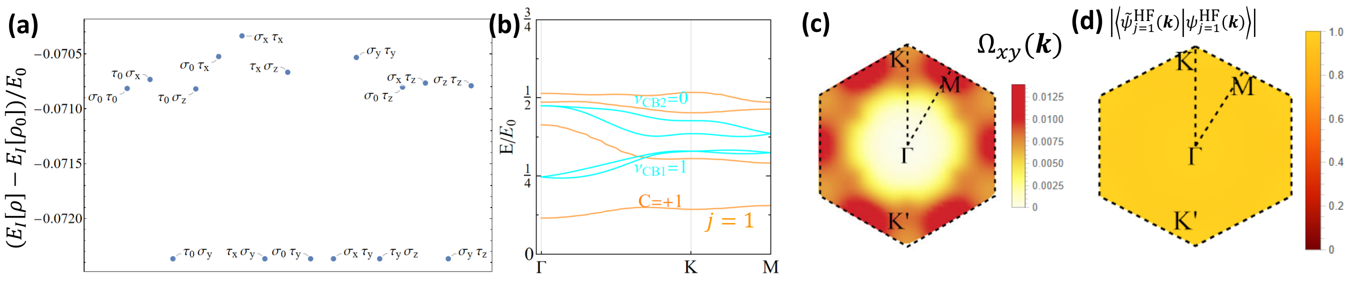

Supplementary Figure 14. (a) The energies of the self-consistent Hartree Fock states with different initial density matrices $\rho^{\rm R}_{ini}(\mathbf{k})$. (b) The spectra (orange) of $H^{\rm HF}(\mathbf{k})$ with $\rho^{\rm R}_{ini}(\mathbf{k})=\sigma_y\tau_0$ and $U(a^{\rm M}_1)=0.13E_0$. The cyan lines are non-interacting spectrum. (c) Berry curvature for the lowest band of $H^{\rm HF}(\mathbf{k})$ in (b). (d) The overlap between the ground states $|\tilde{\psi}^{\rm HF}_{j=1}(\mathbf{k})\rangle$ for four-band Hartree-Fock calculations and the ground states $|\psi^{\rm HF}_{j=1}(\mathbf{k})\rangle$ for two-band Hartree-Fock calculations. Here the calculation is for the four-band model with the parameters $\phi=0,\alpha=0,V_0/E_0=1.2$.

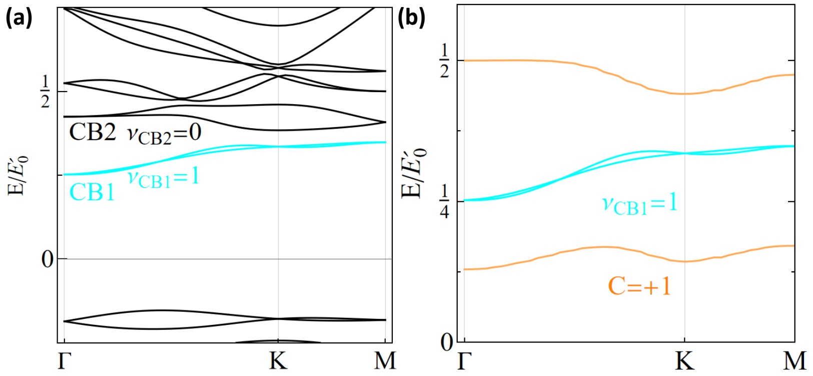

Supplementary Figure 15. (a) Spectrum for the moiré system for smaller moiré unit cells. (b) The spectra (orange) of $H^{\rm HF}$ with $\rho^{\rm R}_y(\mathbf{k})$ for mirror polarized states. Here the calculation is for the two-band model with the parameters $\phi=1/3,\alpha=0.16,V_0/E'_0=1.1$.

and that in Fig. 13(d) for regime B has $C=+1$. Fig. 13(b) and (e) show the distribution of Berry curvature Ω_{xy} in the moiré BZ for regime C and regime B, respectively. Fig. 13(c) and (f) show the projection of $|\tilde{\psi}^{\rm HF}_{j=1}(\mathbf{k})\rangle$ into non-interacting states $|u^{\rm CB1}_{\pm i}(\mathbf{k})\rangle$ and $|u^{\rm CB2}_{\pm i}(\mathbf{k})\rangle$ in regime C and regime B, respectively. One can see that the interacting ground state $|\tilde{\psi}^{\rm HF}_{j=1}(\mathbf{k})\rangle$ has a strong component from $|u^{\rm CB2}_{\pm i}(\mathbf{k})\rangle$, in addition to $|u^{\rm CB1}_{\pm i}(\mathbf{k})\rangle$, due to the strong band mixing in regime C , while only the $|u^{\rm CB1}_{\pm i}(\mathbf{k})\rangle$ part dominates the interacting ground state in regime B . Thus, from Fig. 13, we show that the Coulomb interaction can drive the interacting ground state into a trivial Mott insulator phase [17, 18] via band mixing between CB1 and CB2 when there is inversion symmetry.

For the case $\alpha=0,V_0/E_0=1.2,\phi=0$ without inversion symmetry, the self-consistent solutions are summarized in Fig. 14. As shown in the phase diagram of Fig. 4(b) in the main text, the system stays in the QAH state with $C=+1$ for the realistic Coulomb interaction strength $U(a^{\rm M}_1)=U_0\approx 0.13E_0$, which is quite different from the inversion symmetric case. Here we show more details of this calculation in Fig. 14 for $U(a^{\rm M}_1)=0.13E_0$. Fig. 14(a) shows that the self-consistent solutions with the initial complex density matrices that break the $C_{2z}\mathcal{T}$ still have lower energy. We consider $\sigma_0\tau_y$ as an example and show the energy dispersion of HF bands in Fig. 14(b). The distribution of the Berry curvature Ω_{xy} in the moiré BZ is shown in Fig. 14(c). We further project the interacting ground state of the four-band model, denoted as $|\tilde{\psi}^{\rm HF}_{j=1}(\mathbf{k})\rangle$, into that of the two-band model, denoted as $|\psi^{\rm HF}_{j=1}(\mathbf{k})\rangle$, and find their overlap is almost 1 in the whole moiré BZ, as shown in Fig. 14(d). Thus, the inter-band mixing is negligible in the inversion asymmetric case for $U(a^{\rm M}_1)=U_0\approx 0.13E_0$.

E. Coulomb interaction for smaller moiré unit cells

In this section, we discuss the moiré systems with a smaller moiré lattice constant, $|\mathbf{a}^{\rm M}_1|=14$nm, for a twist angle $\theta=1.0°$. The Coulomb interaction scales inversely with the moiré unit cell length and its strength can be estimated

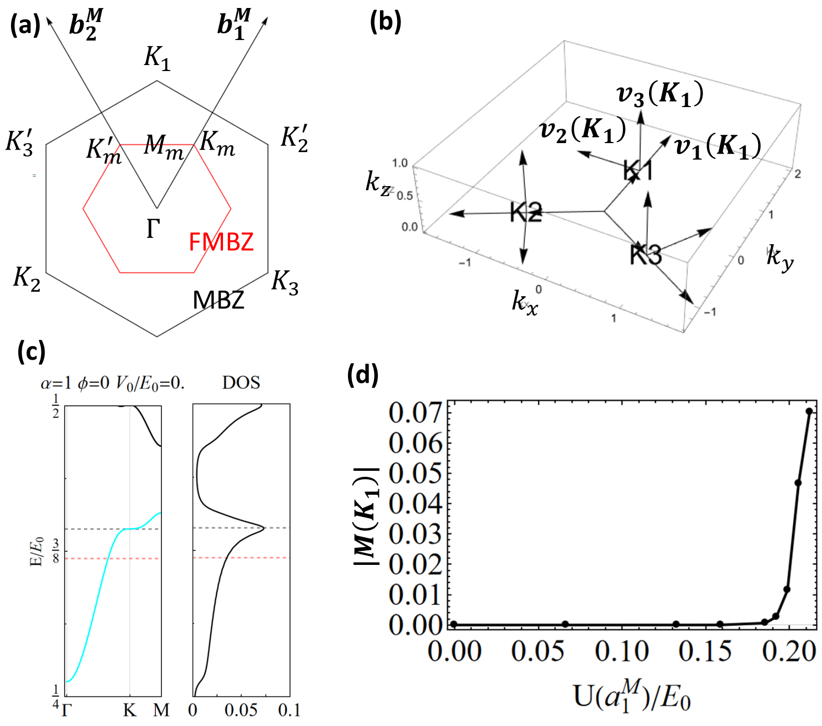

Supplementary Figure 16. (a) The moiré BZ (MBZ) and the folded moiré BZ (FMBZ). (b) The $\mathcal{C}_{3z}$ symmetry adapted basis unit vectors for $\mathbf{M}(\mathbf{Q})$. (c) The single-particle spectrum and density of states (DOS) for CB1 (blue lines). The black dotted line is the Fermi energy with Von-Hove singularities while the red dotted line is the Fermi energy for half filling of CB1. (d) The magnetic texture order parameters $|\mathbf{M}(\mathbf{K}_1)|$ versus different Coulomb interaction strength $U(\mathbf{a}_1^M)/E_0$.

as $U(\mathbf{a}_1^{\mathrm{M}}) = 10$ meV.

The spectrum is shown in Fig. 15(a). The parameters for the spectrum are $\Delta_1 = 14$meV, $\phi = 1/3$, and $m_0 = 30$meV. The energy scale is $E_0' = v|\mathbf{b}_1^{\mathrm{M}}| = 77$meV. The bandwidth of CB1 is 7.4meV and the direct gap between CB1 and CB2 is 4meV. The ratio between $U(\mathbf{a}_1^{\mathrm{M}})$ and bandwidth is smaller for the smaller $|\mathbf{a}_1^{\mathrm{M}}|$. When the Coulomb interaction is considered for CB1 with the density matrix $\rho_y^{\mathrm{R}}(\mathbf{k})$ as shown in Fig. 15(b), the mirror polarized states with $C = +1$ can be induced.

F. Spin density wave

In this section, we discuss the nonuniform magnetic order parameters within the Hartree-Fock mean field approximation following Ref. [19].

To construct the nonuniform magnetic order parameters, we first write down the Coulomb interaction in Eq. (53) using spin operators under the Hartree Fock approximation as

$$
\begin{aligned}
H_{\mathrm{I}} = & \sum_{\mathbf{q},\mathbf{G}} V(\mathbf{q}+\mathbf{G}) \Big(\sum_{\mathbf{k}_1,\mathbf{G}_1,\alpha_1} f^\dagger_{\alpha_1}(\mathbf{k}_1+\mathbf{G}_1+\mathbf{q}+\mathbf{G}) f_{\alpha_1}(\mathbf{k}_1+\mathbf{G}_1) \sum_{\mathbf{k}_2,\mathbf{G}_2,\alpha_2} \langle f^\dagger_{\alpha_2}(\mathbf{k}_2+\mathbf{G}_2-\mathbf{q}-\mathbf{G}) f_{\alpha_2}(\mathbf{k}_2+\mathbf{G}_2)\rangle \Big) \\
& - \frac{1}{2} \sum_{\mathbf{q},\mathbf{G}} \sum_{\mathbf{k}_1,\mathbf{G}_1,\alpha_1,\alpha_1'} \sum_{\mathbf{k}_2,\mathbf{G}_2,\alpha_2,\alpha_2'} V(\mathbf{k}_1+\mathbf{G}_1-\mathbf{k}_2-\mathbf{G}_2+\mathbf{q}+\mathbf{G}) \\
& \Big(\sum_i f^\dagger_{\alpha_1}(\mathbf{k}_1+\mathbf{G}_1+\mathbf{q}+\mathbf{G}) (\tau_0 s_i)_{\alpha_1,\alpha_1'} f_{\alpha_1'}(\mathbf{k}_1+\mathbf{G}_1) \langle f^\dagger_{\alpha_2}(\mathbf{k}_2+\mathbf{G}_2-\mathbf{q}-\mathbf{G}) (\tau_0 s_i)_{\alpha_2,\alpha_2'} f_{\alpha_2'}(\mathbf{k}_2+\mathbf{G}_2)\rangle \\
& + f^\dagger_{\alpha_1}(\mathbf{k}_1+\mathbf{G}_1+\mathbf{q}+\mathbf{G}) \delta_{\alpha_1,\alpha_1'} f_{\alpha_1'}(\mathbf{k}_1+\mathbf{G}_1) \langle f^\dagger_{\alpha_2}(\mathbf{k}_2+\mathbf{G}_2-\mathbf{q}-\mathbf{G}) \delta_{\alpha_2,\alpha_2'} f_{\alpha_2'}(\mathbf{k}_2+\mathbf{G}_2)\rangle \Big) \\
& - E_{\mathrm{C}},
\end{aligned}
\tag{81}
$$

where τ and s are Pauli matrices for the top/bottom surfaces and spin degrees of freedom as in Eq. (1) of the main

text, respectively. $i = x, y, z$. E_C is the condensation energy. Eq. (81) can be restored to Eq. (59) by projecting the electron operators into the eigenstate basis (Eq. 48), imposing $q = 0$ and making use of the equality

$$\sum_i s^i_{\alpha_1,\alpha_1'} s^i_{\alpha_2,\alpha_2'} = 2\delta_{\alpha_1,\alpha_2'}\delta_{\alpha_1',\alpha_2} - \delta_{\alpha_1,\alpha_1'}\delta_{\alpha_2,\alpha_2'}. \tag{82}$$

The nonuniform magnetic order parameter or spin density wave has the form of $M_i(\mathbf{Q}) = \langle \mathcal{S}_i(\mathbf{Q})\rangle$ in Ref. [19] with

$$\begin{aligned}\mathcal{S}_i(\mathbf{Q}) &= \sum_{\mathbf{k}_1,\mathbf{G}_1,\alpha_1,\alpha_1'} f^\dagger_{\alpha_1}(\mathbf{k}_1+\mathbf{G}_1+\mathbf{Q})(\tau_0 s_i)_{\alpha_1,\alpha_1'} f_{\alpha_1'}(\mathbf{k}_1+\mathbf{G}_1) \\ &= \sum_{\mathbf{k}_1,n,n'} c^\dagger_n(\mathbf{k}_1+\mathbf{Q})\Lambda^{s_i}_{n,n'}(\mathbf{k}_1+\mathbf{Q},\mathbf{k}_1)c_{n'}(\mathbf{k}_1),\end{aligned} \tag{83}$$

where we have used the eigen-state projection Eq. 48 in the second line. As in Ref. [19], $\mathbf{Q}$ takes the values of $\mathbf{K}_1, \mathbf{K}_2, \mathbf{K}_3, \mathbf{K}_1', \mathbf{K}_2', \mathbf{K}_3'$ shown in Fig. 16(a) that connects $\mathbf{K}$ and $\mathbf{K}'$ in the moiré BZ because of the large density of states around $\mathbf{K}$ and $\mathbf{K}'$ Fig. 16(c). $\Lambda^{s_i}_{n,n'}(\mathbf{k}+\mathbf{Q},\mathbf{k})$ is the form factor of the spin operator defined as

$$\Lambda^{s_i}_{n,n'}(\mathbf{k}+\mathbf{Q},\mathbf{k}) = \sum_{\mathbf{G},\alpha,\alpha'} u^{n*}_{\mathbf{G},\alpha}(\mathbf{k}+\mathbf{Q})(\tau_0 s_i)_{\alpha,\alpha'} u^{n'}_{\mathbf{G},\alpha'}(\mathbf{k}) \tag{84}$$

and satisfies

$$\Lambda^{s_i}_{n,n'}(\mathbf{k}+\mathbf{Q},\mathbf{k}) = \Lambda^{s_i}_{n',n}(\mathbf{k},\mathbf{k}+\mathbf{Q}) = \Lambda^{s_i}_{n,n'}(\mathbf{k}+\mathbf{Q}+\mathbf{G},\mathbf{k}+\mathbf{G}). \tag{85}$$

A real order parameter $\mathbf{M}(\mathbf{r}) = \sum_{\mathbf{Q}} e^{i\mathbf{Q}\cdot\mathbf{r}}\mathbf{M}(\mathbf{Q})$ in the real space requires

$$\mathbf{M}(\mathbf{Q}) = \mathbf{M}^*(-\mathbf{Q}). \tag{86}$$

The order parameters have the $\mathcal{C}_{3z}$ symmetry by

$$\mathcal{R}_{3z}\mathbf{M}(\mathbf{Q}) = \mathbf{M}(\mathcal{R}_{3z}\mathbf{Q}) \tag{87}$$

where $\mathcal{R}_{3z}$ is the rotation matrix about z axis. $\mathcal{C}_{3z}$ symmetry adapted basis unit vectors for $\mathbf{M}(\mathbf{Q})$ is shown in Fig. 16(b) satisfying

$$\mathcal{R}_{3z}\mathbf{v}^s_\mu(\mathbf{Q}) = \mathbf{v}^s_\mu(\mathcal{R}_{3z}\mathbf{Q}) \tag{88}$$

where $\mu = 1, 2, 3$ and

$$v^s_1 = \mathbf{Q}/|\mathbf{Q}|, v^s_2 = \hat{z}\times\mathbf{Q}/|\mathbf{Q}|, v^s_3 = \hat{z}. \tag{89}$$

$\hat{z}$ is the unit vector in the z direction. We decompose $\mathbf{M}(\mathbf{Q})$ into basis unit vectors as

$$\mathbf{M}(\mathbf{Q}) = \sum_\mu M_\mu(\mathbf{Q})\mathbf{v}^s_\mu(\mathbf{Q}) = \sum_\mu M_\mu \mathbf{v}^s_\mu(\mathbf{Q}). \tag{90}$$

Here we only consider the non-uniform order parameter with $\mathcal{C}_{3z}$ symmetry, so M_μ is independent of $\mathbf{Q}$. The interaction Hamiltonian in the $\mathbf{M}(\mathbf{Q})$ order parameter channel is

$$H_{\mathrm{I}}[\mathbf{M}] = -\frac{1}{2}V_s\sum_{\mathbf{Q},i}\mathcal{S}_i(\mathbf{Q})M_i(-\mathbf{Q}) - E_{\mathrm{C}}[\mathbf{M}] \tag{91}$$

with

$$E_{\mathrm{C}}[\mathbf{M}] = -\frac{1}{4}V_s\sum_{\mathbf{Q},i}M_i(\mathbf{Q})M_i(-\mathbf{Q}). \tag{92}$$

The following approximation is also adopted

$$V(\mathbf{k}_1+\mathbf{G}_1-\mathbf{k}_2-\mathbf{G}_2+\mathbf{Q}) \approx V(\mathbf{Q}) = V_s. \tag{93}$$

The last equation holds because $V(\mathbf{Q})$ only depends on $|\mathbf{Q}|$ in Eq. (52) and are same for $\mathbf{Q} = \mathbf{K}_1, \mathbf{K}_2, \mathbf{K}_3, \mathbf{K}'_1, \mathbf{K}'_2, \mathbf{K}'_3$.

Because $\mathbf{M}(\mathbf{Q})$ breaks the moiré translation symmetry, it folds moiré BZ as shown in Fig. 16(a) and couples states at $\boldsymbol{\kappa}, \boldsymbol{\kappa}+\mathbf{K}_1, \boldsymbol{\kappa}+\mathbf{K}'_1$ where $\boldsymbol{\kappa}$ is the momentum in the folded moiré BZ (FMBZ). The explicit form of the Hartree-Fock mean field Hamiltonian in the basis $C(\boldsymbol{\kappa}) = (c_{n_1}(\boldsymbol{\kappa}), c_{n_2}(\boldsymbol{\kappa}+\mathbf{K}_1), c_{n_3}(\boldsymbol{\kappa}+\mathbf{K}'_1))$ is

$$H^{\mathrm{HF}}[\mathbf{M}](\boldsymbol{\kappa}) = \begin{pmatrix} E_0^{n_1}(\boldsymbol{\kappa}) & 0 & 0 \\ 0 & E_0^{n_2}(\boldsymbol{\kappa}+\mathbf{K}_1) & 0 \\ 0 & 0 & E_0^{n_3}(\boldsymbol{\kappa}+\mathbf{K}'_1) \end{pmatrix} - \frac{1}{2}V_s \sum_i \sum_{\mathbf{Q}\in(\mathbf{K}_1,\mathbf{K}_2,\mathbf{K}_3)}$$
$$\Big[M^i(-\mathbf{Q}) \begin{pmatrix} 0 & 0 & \Lambda^{s_i}_{n_1,n_3}(\boldsymbol{\kappa}+\mathbf{Q}+\mathbf{K}'_1, \boldsymbol{\kappa}+\mathbf{K}'_1) \\ \Lambda^{s_i}_{n_2,n_1}(\boldsymbol{\kappa}+\mathbf{Q}, \boldsymbol{\kappa}) & 0 & 0 \\ 0 & \Lambda^{s_i}_{n_3,n_1}(\boldsymbol{\kappa}+\mathbf{Q}+\mathbf{K}_1, \boldsymbol{\kappa}+\mathbf{K}_1) & 0 \end{pmatrix} \tag{94}$$
$$+M^i(\mathbf{Q}) \begin{pmatrix} 0 & \Lambda^{s_i}_{n_1,n_2}(\boldsymbol{\kappa}-\mathbf{Q}+\mathbf{K}_1, \boldsymbol{\kappa}+\mathbf{K}_1) & 0 \\ 0 & 0 & \Lambda^{s_i}_{n_2,n_3}(\boldsymbol{\kappa}-\mathbf{Q}+\mathbf{K}'_1, \boldsymbol{\kappa}+\mathbf{K}'_1) \\ \Lambda^{s_i}_{n_3,n_1}(\boldsymbol{\kappa}-\mathbf{Q}, \boldsymbol{\kappa}) & 0 & 0 \end{pmatrix} \Big].$$

As

$$(M^i(-\mathbf{Q})\Lambda^{s_i}_{n_2,n_1}(\boldsymbol{\kappa}+\mathbf{Q},\boldsymbol{\kappa}))^* = M^i(\mathbf{Q})\Lambda^{s_i}_{n_1,n_2}(\boldsymbol{\kappa},\boldsymbol{\kappa}+\mathbf{Q}) = M^i(\mathbf{Q})\Lambda^{s_i}_{n_1,n_2}(\boldsymbol{\kappa}-(\mathbf{Q}-\mathbf{K}_1),\boldsymbol{\kappa}+\mathbf{K}_1), \tag{95}$$

H^{HF} is hermitian. In the last equation, we have used Eq. (85) and the fact that $\mathbf{Q}-\mathbf{K}_1$ for $\mathbf{Q} \in (\mathbf{K}_1, \mathbf{K}_2, \mathbf{K}_3)$ is a moiré reciprocal lattice vector vector. To perform the self-consistent calculations, we choose the initial condition of the magnetic order parameters as

$$M_\mu = (0.01, 0, 0.01i), \tag{96}$$

corresponding to the magnetic meron lattice discussed in Ref. [19]. With $H^{\mathrm{HF}}[\mathbf{M}]$ and the initial condition, the self-consistent calculations are done until

$$|\mathbf{M}(\mathbf{K}_1) - \tilde{\mathbf{M}}(\mathbf{K}_1)| < 0.01|\mathbf{M}(\mathbf{K}_1)| \tag{97}$$

with $\mathbf{M}(\mathbf{K}_1), \tilde{\mathbf{M}}(\mathbf{K}_1)$ as the order parameters for ath and $a+1$th iterations, respectively.

We next discuss the Hartree-Fock mean-field solutions at half-filling of CB1 for the inversion symmetric case. In the single-particle spectrum, there are Von-Hove singularities near $\mathbf{K}$ and $\mathbf{K}'$ at filling 0.8 of CB1, which is away from the half filling for our self-consistent calculations, as shown in Fig. 16(c). The Von-Hove singularities correpsond to a peak in density of states (DOS) calculated by

$$DOS(E) = \sum_n \int \frac{\mathrm{d}^2\mathbf{k}}{(2\pi)^2} \frac{2\gamma}{(E - E_n^0(\mathbf{k}))^2 + \gamma^2} \tag{98}$$

with broadening $\gamma = 0.005E_0$. Here we choose the Lorentz form instead of δ function for the numerical calculations. The critical Coulomb interaction strength for nonzero magnetic meron texture is around $0.18E_0$, as shown in Fig. 16(d), which is much larger than the critical interaction $0.05E_0$ for the QAH phase discussed in the main text. In the moiré TI system, the Coulomb interaction is estimated to be $0.13E_0$, which is smaller than that for magnetic meron texture but larger than that of QAH phase. Thus, we conclude that the QAH state, instead of magnetic meron texture, is the ground state at half filling of the lowest conduction band. We also expect that the magnetic meron texture in Ref Ref. [19] can be stabilized for the filling around 0.8 of the CB1.

Supplementary Note 3. COMPUTATIONAL METHODS FOR DFT CALCULATIONS AND MOIRÉ LATTICE

The DFT calculations were performed with the Vienna Ab initio Simulation Package (VASP)[20]. The exchange-correlation functional was chosen as the Perdew-Burke-Ernzerhof type generalized-gradient approximation[21] and the projector-augmented-wave method was used for the core-electron potentials[22, 23]. The energy cutoff was set as

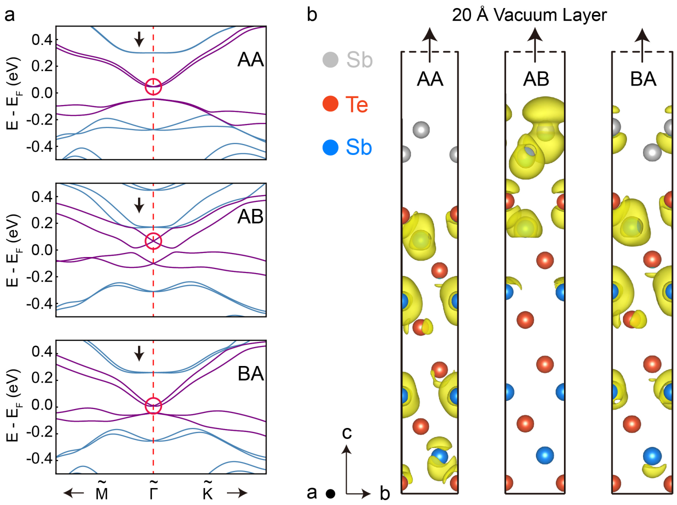

Supplementary Figure 17. Illustration of the electronic structures of the three high-symmetric stacking structures. (a) Band structures of the high-symmetric stacking structures (AA, AB, and BA). The black arrows mark the bulk states. (b) The charge density distribution on the conduction band edge at the $\tilde{\Gamma}$ point (red circles in (a)) in the real space of the corresponding slab models. The iso-surface is set as 0.0005 eÅ^{-3}.

340 eV for all calculations. The convergence criterion was set as 10^{-5} eV for self-consistent electronic calculations and the k-point meshes were set as $13 \times 13 \times 1$ to sample the Brillouin zone. We used the DFT-D3 method[24] to correctly describe the van der Waals interactions.

As discussed in the main text and the next section Supplementary Note 4, the moiré potential $\Delta(\mathbf{r})$ in the twisted Sb_2/Sb_2Te_3 hetero-structures could be obtained from the uniform potential $\tilde{\Delta}(\mathbf{d_R})$ for the hetero-structure with a uniform shift $\mathbf{d_R}$ between the Sb_2 layer and Sb_2Te_3 layer. Therefore, we will describe below our first principles calculations of the band structures with different stacking configurations that correspond to different shifting vectors $\mathbf{d_R}$. As shown in Fig. 19(a), the heterostructure model with the specific stacking contains 1×1 Sb_2 monolayer and 1×1 2QL Sb_2Te_3 thin film. A vacuum layer with 20 Å was added along the z direction to avoid the interaction between adjacent slabs. Because the uniform potential $\tilde{\Delta}(\mathbf{d_R})$ is induced by coupling between Sb_2 monolayer and Sb_2Te_3 thin films, we fixed the value of the van der Waals gap inside 2QL Sb_2Te_3 thin film as the bulk value (2.708 Å), and then let Sb_2 monolayer and its neighboring atoms fully relax until the calculated forces are smaller than 0.01 eV/Å. In Fig. 5 of the main text, we plot the relaxed lattices and corresponding band structures for heterostructures with AA, AB, and BA stacking, whose interlayer distances between Sb_2 monolayer and 2QL Sb_2Te_3 thin film are 3.92 Å, 2.81 Å, and 2.91 Å respectively. The charge density distribution for the conduction band edge at the $\tilde{\Gamma}$ point is also plotted in Fig. 17 for Sb_2/Sb_2Te_3 with different stackings. For such an ultra-thin film, the strong quantum confinement will dominate the electronic structures around the Fermi level, and these states at conduction band edge are all contributed from the quantum well states that originate from the strong hybridization of the top and bottom surface states [25]. These quantum-well states also couple with states from Sb_2 monolayer. Compared with AA and BA stackings, there is relatively large distribution on Sb_2 monolayer for conduction band edge state at the $\tilde{\Gamma}$ point and the coupling strength between Sb bilayer with Sb_2Te_3 in AB stacking is largest. For the AA and BA stacking, the conduction band edge states at the $\tilde{\Gamma}$ point are mainly localized in the 2QL Sb_2Te_3 .

The strain on the Sb_2Te_3 thin film by fixing the atoms in the green region of Fig. 19(a) of the Sb_2Te_3 is negligible after comparing with the fully relaxed structure of the slab model. The AA stacking is taken as an example. The structure by the relaxation method in the paper is listed in Tab.IV and marked as AA-I. The full relaxation model is to fully relax all atoms' coordinates with the fixed volume of the unit cell and the obtained structure is marked as AA-II. The relative displacement of corresponding atoms in both AA-I and AA-II models represents the influence of the induced strain on the Sb_2Te_3 thin film. As shown in Tab.IV, the maximum value for the difference of atom positions is 0.009 Å, which is small enough to be neglected.

Besides the stacking configurations shown in the main text, other stacked heterostructures can be also found in the moiré pattern (see Fig. 5 in the main text). In order to calculate moiré potential $\Delta(\mathbf{r})$ accurately (see Section Supplementary Note 4 below), we consider extra nine stacked configurations, as shown in Fig. 19(b-d), which are located in the intermediate regions among AA, AB, and BA stackings, named AAmAA-X (Fig. 19(b)), AAmAB-X (Fig. 19(c)), and AAmBA-X (Fig. 19(d)) with X = I, II, III. We take the heterostructure models with AAmAA stackings as an example to show their lattices in details and the other intermediate stacked configurations could be

Atom	AA-I			AA-II			Δx (Å)	Δy (Å)	Δz (Å)
Index	x (Å)	y (Å)	z (Å)	x (Å)	y (Å)	z (Å)			
1	2.128	3.691	22.644	2.129	3.691	22.643	0.001	0.000	0.001
2	0.000	2.462	12.430	0.000	2.461	12.430	0.000	0.000	0.000
3	2.132	3.693	5.000	2.132	3.693	5.003	0.000	0.000	0.003
4	2.132	1.231	15.210	2.131	1.230	15.214	0.001	0.000	0.004
5	2.132	1.231	8.715	2.132	1.231	8.714	0.000	0.000	0.000
6	-0.003	2.460	18.931	-0.002	2.461	18.931	0.001	0.000	0.000
7	0.001	0.000	16.939	0.000	0.000	16.940	0.001	0.000	0.001
8	0.000	2.462	6.729	0.000	2.462	6.720	0.000	0.000	0.009
9	2.132	3.692	10.700	2.132	3.693	10.703	0.000	0.000	0.003
10	2.130	1.229	20.919	2.130	1.229	20.917	0.000	0.000	0.002
11	2.187	1.258	28.156	2.187	1.259	28.157	0.000	0.000	0.001
12	0.055	0.028	26.564	0.055	0.028	26.565	0.000	0.000	0.001

Supplementary Table IV. Cartesian coordinates of atoms in relaxed structures (AA-I/II) and the difference of the coordinates of the corresponding atoms.

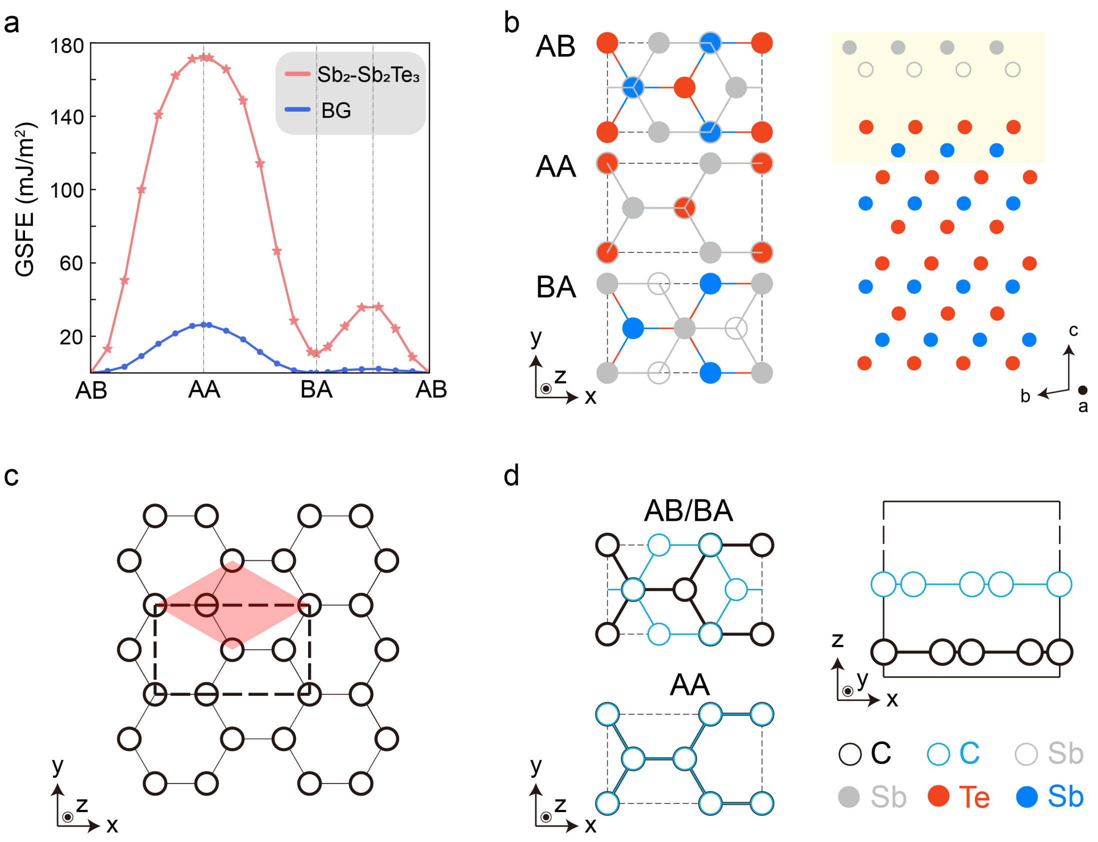

Supplementary Figure 18. The illustration of the generalized stacking-fault energy for both bilayer graphene and Sb_2/2QL Sb_2Te_3 model. (a) The variation of the GSFE of both bilayer graphene and Sb_2/2QL Sb_2Te_3 structures along with the transition of the stacking orders. (b) The high-symmetric stacking configurations of the Sb_2/2QL Sb_2Te_3 model (left panel) and the side view of the Sb_2/2QL Sb_2Te_3 heterostructure (right panel). Only atoms in the light-yellow areas are plotted in the left panel to show every stacking order clearly. (c) The illustration of the construction of the supercells (dashed rectangle) from the hexagonal primitive cells (red). (d) The high-symmetric stacking configurations of bilayer graphene (left panel) and the side view of the bilayer graphene (right panel).

obtained by using the same method. There are three types of AAmAA stacked structures in the moiré pattern, corresponding to the shifting vector $\mathbf{d}_R$ as $\tilde{\mathbf{a}}_1/2 + 0\tilde{\mathbf{a}}_2$, $0\tilde{\mathbf{a}}_1 - \tilde{\mathbf{a}}_2/2$, and $\tilde{\mathbf{a}}_1/2 + \tilde{\mathbf{a}}_2/2$ for AAmAA-I, AAmAA-II, and AAmAA-III respectively. These three configurations are related by $\mathcal{C}_{3z}$ rotation, and thus we only need to calculate the electronic structure for one of them. On the other hand, because these intermediate stacked structures are not in the local minimum of the potential energy surface, we only relax the z direction coordinate while fix the x and y coordinates of the Sb_2 monolayer (see Fig. 19(a)). The corrugation effect, which is crucial for predicting the correct band structure in twisted bilayer graphene[26–28], is taken into account after this lattice relaxation process. Using the same method, we obtained the relaxed intermediate stacked heterostructures for AAmAB-X and AAmBA-X (X = I, II, III). The interlayer distances between Sb_2 monolayer and 2QL Sb_2Te_3 thin film are 2.94 Å, 3.38 Å, and 3.42 Å for AAmAA, AAmAB, and AAmBA stacked heterostructures respectively and the related band structures are shown in Fig. 19(e) correspondingly.

Although the corrugation effect is neglected in this calculation, the Sb_2/2QL Sb_2Te_3 heterostructure is easier to relax at the moiré scale with the small twisted angle compared to the twisted bilayer graphene (TBG). In the TBG, if the twist angle is smaller than $0.5°$, evident lattice reconstruction will occur where the AA stacking regimes decrease while the AB/BA stacking order areas increase [29]. Such a phenomenon results from the competition between the energy gain from stacking configuration transitions and the elastic energy cost induced by the lattice deformation [30]. For a twisted system, if more energy gain is obtained during the transition from a metastable stacking to the most stable stacking, such twisted system will prefer to relax at the moiré scale with the small twisted angle. Furthermore, to be similar, if less energy cost is expended during the lattice deformation process, the target twisted system will also prefer to relax at the moiré scale. Therefore, the possibility of the relaxation for the twisted Sb_2/2QL Sb_2Te_3 heterostructure in moiré scale can be evaluated from two aspects. One is the energy gain from the transition among various stacking configurations, while the other is the elastic energy cost during the lattice deformation. The generalized stacking-fault energy (GSFE) can be used to evaluate the energy gain in the stacking configuration transitions, which is defined as the difference of energy density between metastable stacking structures and the most stable stacking structure [31, 32]. Following the method reported in Ref. [32], we firstly build $\sqrt{3} \times 1$ supercells (dashed rectangle in Fig. R2c) for both bilayer graphene and Sb_2/2QL Sb_2Te_3 and choose the AB stacking configuration as the initial stacking order. The top graphene layer (blue lattice in 18(d) and the Sb_2 monolayer (grey lattice in 18(b)) are moved along the x direction (see 18(b)(d)) to construct a series of stacking configurations and calculate the GSFE accordingly. Calculated results are shown in 18(a). In the left panels of 18(b)(d), we plot three high-symmetric stacking configurations whose GSFE correspond to the extreme points in 18(a). The maximum of the GSFE of the Sb_2/2QL Sb_2Te_3 system is around 172 $mJ\ m^{-2}$, which is almost 7 times larger than that of the bilayer graphene system (26 $mJ\ m^{-2}$). Therefore, based on DFT calculations, the energy gain from the transition among various stacking orders in the Sb_2/2QL Sb_2Te_3 system will be much larger than that in the bilayer graphene. For elastic deformation in the van der Waals stacking structures, the elastic energy (U) can be defined as:

$$U = \sum_{l=t,b} \left(\frac{1}{2}\frac{E_l v_l}{(1+v_l)(1-v_l)}(\sigma_{ii}^{(l)})^2 + \frac{1}{2}\frac{E_l}{1+v_l}(\sigma_{ij}^{(l)})^2\right) \tag{99}$$

Here, index l refers to the top layer (t) or bottom layer (b). E_l and v_l are Young's moduli and Poisson ratios for each layer. Moreover, $\sigma^{(l)}$ refers to strain tensors for each layer [30]. It is known that the graphene has the largest in-plane Young's modulus. Therefore, under the same strain field, the elastic deformation energy of the bilayer graphene system would be much larger than that of the Sb_2/2QL Sb_2Te_3 heterostructure. This result indicates that in-plane lattice deformation is easier in Sb_2/2QL Sb_2Te_3 slab model. To conclude, compare with TBG system, the twisted Sb_2/2QL Sb_2Te_3 heterostructure is easier to relax at the moiré scale with the small twisted angle due to the larger GFSE and smaller elastic energy cost during the lattice deformation.

Supplementary Note 4. MOIRÉ POTENTIALS FROM THE FITTING TO THE DFT BAND STRUCTURE

In this section, we discuss the method to obtain Moire potential $\Delta(\mathbf{r})$ in Eq. 1 of the main text from the above DFT calculation in Sec. Supplementary Note 3 [33].

We first consider the effective Hamiltonian H^{DFT} in Eq. (3) of the main text. Compared to the original Hamiltonian H_0 in Eq. 1, the spatially dependent moiré potential term $\Delta(\mathbf{r})$ is changed to a uniform potential term $\tilde{\Delta}(\mathbf{d}_R)$ for a fixed $\mathbf{d}_R$ that describes the relative shift between Sb_2 and Sb_2Te_3 layers. H^{DFT} describes the effective model for the hetero-structure with a uniform shift between two atomic layers, and different values of the shifting vector $\mathbf{d}_R$ describe different stacking configurations. Thus, we can use the energy dispersion of H^{DFT} to fit to that from the DFT calculations.

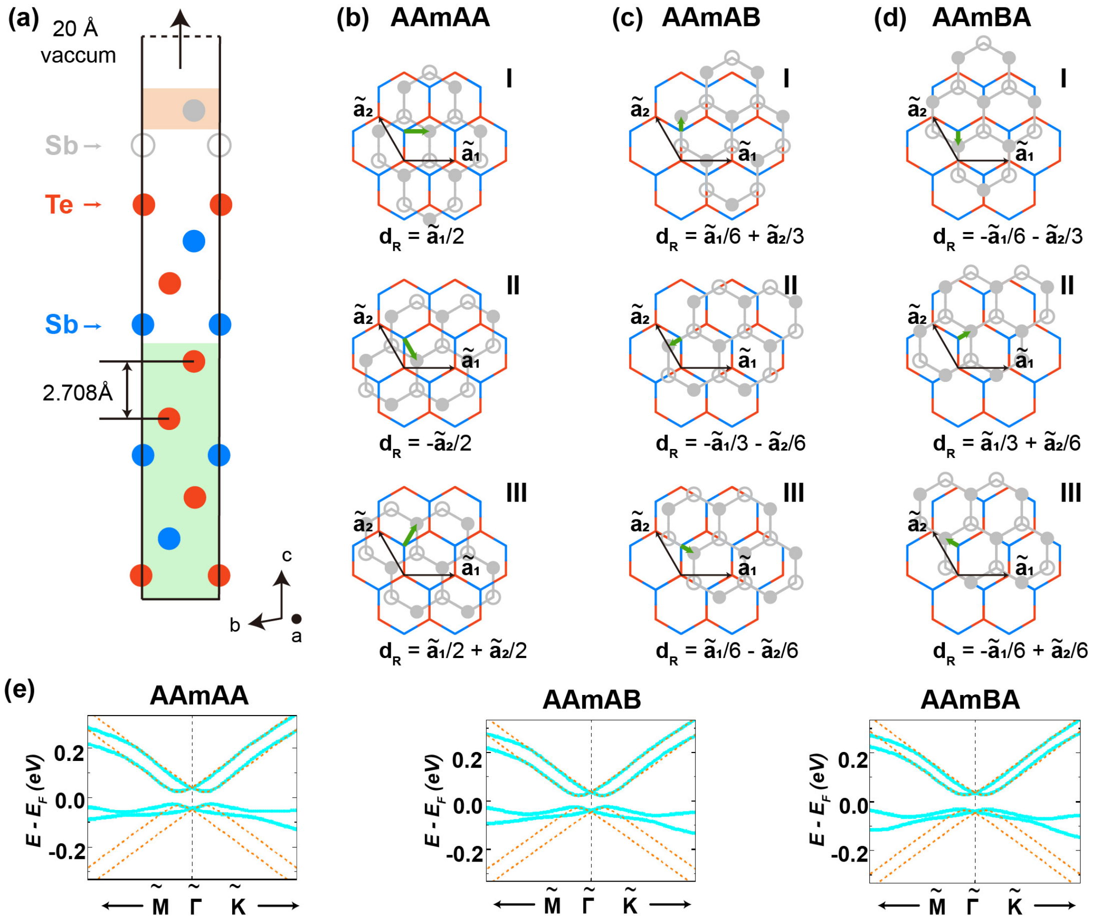

Supplementary Figure 19. (a) The side view of the Sb_2/Sb_2Te_3 heterostructure with AA stacking. The solid black lines mark the unitcell used in DFT calculations. Sb atoms in Sb_2 monolayer are marked as gray, Sb atoms and Te atoms in Sb_2Te_3 films are marked as red and blue. The atoms in the region with the green background are frozen when we relax the lattice structures, the x and y coordinates of Sb atoms in the region with the yellow background are fixed. The value of the van der Waals gap inside 2QL Sb_2Te_3 films is 2.078Å. (b-d) The top view for heterostructures with the stacking of AAmAA-X, AAmAB-X, and AAmBA-X (X = I, II, III). Corresponding $\mathbf{d}_R$ is shown by the green arrows. The black arrows show the lattice vector of Sb_2 monolayer. (e) Calculated band structures (cyan lines) of the heterostructure with the stacking of AAmAA, AAmAB, and AAmBA, respectively. The Fermi levels are set as zero. The orange dashed lines are fitted spectra by the Hamiltonian Eq. (100) for a uniform shift.

The effective Hamiltonian $\hat{H}^{\mathrm{DFT}}(\mathbf{d}_R)$ is then given by

$$\langle \mathbf{k}_1, \beta_1 | \hat{H}^{\mathrm{DFT}}(\mathbf{d}_R) | \mathbf{k}_2, \beta_2 \rangle = \delta(\mathbf{k}_1 - \mathbf{k}_2)\left(\begin{pmatrix} h_D^t(\mathbf{k}_1) & m s_0 \\ m s_0 & h_D^b(\mathbf{k}_1) \end{pmatrix} + \begin{pmatrix} \tilde{\Delta}(\mathbf{d}_R) s_0 & 0 \\ 0 & \alpha \tilde{\Delta}(\mathbf{d}_R) s_0 \end{pmatrix} \right) = \delta(\mathbf{k}_1 - \mathbf{k}_2) H^{\mathrm{DFT}}(\mathbf{k}_1, \mathbf{d_R}), \tag{100}$$

where $H^{\mathrm{DFT}}(\mathbf{k}, \mathbf{d_R})$ is just Eq.(4) in the main text, $h_D^{t/b}(\mathbf{k})$ are the top/bottom Dirac surface states same as Eq. (1) of the main text, s_0 are the identical matrix in spin space, m is the tunnelling between two surfaces, and α captures the difference in the potentials on two surfaces created by the Sb_2 layer. $|\mathbf{k}, \beta\rangle$ is the atomic Bloch states for the Sb_2Te_3 and Sb_2 lattice with a constant shift. $\beta_{1,2} = 1, ..., 4$ represents both the spin and layer degrees of freedom. The spectra of this model are given by

$$E_{\eta,\xi}^{\mathrm{DFT}}(\mathbf{k}, \mathbf{d}) = \frac{1+\alpha}{2}\tilde{\Delta}(\mathbf{d}) + \eta\sqrt{m^2 + \left(\frac{1-\alpha}{2}\tilde{\Delta}(\mathbf{d}) + \xi v^2 k^2)\right)^2}. \tag{101}$$

with $\eta = \pm, \xi = \pm$. By fitting $E_{\eta,\xi}^{\mathrm{DFT}}(\mathbf{k}, \mathbf{d})$ to the spectrum calculated from DFT in Fig.4(b) of the main text and Fig. 19(f), the parameters α, $\tilde{\Delta}(\mathbf{d})$, v and m in the model Hamiltonian can be obtained and documented in Tab.V for

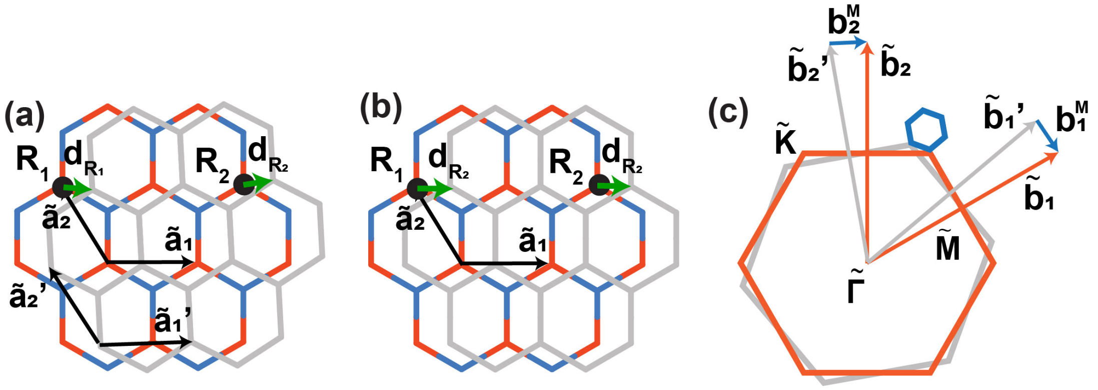

Supplementary Figure 20. (a) The lattice structures for the twisted Sb_2 (gray) on top of Sb_2Te_3 (blue and orange) at a location same as Fig.4(c) of the main text. $\tilde{\mathbf{a}}_{1,2}$ are primitive lattice vectors for the Sb_2Te_3 layer and $\tilde{\mathbf{a}}'_{1,2}$ are primitive lattice vectors for Sb_2 layer. $\mathbf{d}_{\mathbf{R}_{1,2}}$ are the local lattice shifts at sites $\mathbf{R}_{1,2}$. (b) Commensurate Sb_2 (gray) on top of Sb_2Te_3 with a constant shift $\mathbf{d}_{\mathbf{R}_2}$ as an approximation for the local lattice structures in (a). (c) BZs for Sb_2Te_3 (orange), Sb_2 (gray), and moiré superlattice (blue). $\tilde{\mathbf{b}}_{1,2}$, $\tilde{\mathbf{b}}'_{1,2}$, $\tilde{\mathbf{b}}^{\mathrm{M}}_{1,2}$ are reciprocal lattice vectors for for Sb_2Te_3, Sb_2, moiré superlattice, respectively.

Stacking	$\mathbf{d}_\mathbf{R}$	m (eV)	$\frac{(1-\alpha)}{2}\tilde{\Delta}(\mathbf{d}_\mathbf{R})$ (eV)	v (eV Å^{-1})	$\frac{(1+\alpha)}{2}\tilde{\Delta}(\mathbf{d}_\mathbf{R})$ (eV)
AA	0	0.0457	0.0184	3.27	0.000016
AB	$\frac{\tilde{a}_1}{3}+\frac{2\tilde{a}_2}{3}$	0.0193	0.0688	2.3	-0.00487
BA	$\frac{2\tilde{a}_1}{3}+\frac{\tilde{a}_2}{3}$	0.0253	0.0207	3.13	-0.0182
AAmAA	$\frac{\tilde{a}_1}{2}$	0.0266	0.0383	3.05	-0.00452
AAmAB	$\frac{\tilde{a}_1}{6}+\frac{\tilde{a}_2}{3}$	0.0242	0.035	3.	-0.00461
AAmBA	$\frac{5\tilde{a}_1}{6}+\frac{2\tilde{a}_2}{3}$	0.0308	0.0297	3.08	-0.00394

Supplementary Table V. A summary of fitting parameters in the effective Hamiltonian Eq. (100) by comparison of DFT spectra of different stackings and the eigenenergies of $\hat{H}^{\mathrm{DFT}}$.

different stackings of two layers. From the three-fold rotation symmetry C_3 of the underlying lattice, one can obtain

$$\langle \mathbf{k}, \beta_1 | \hat{H}^{\mathrm{DFT}}(\mathbf{d}) | \mathbf{k}, \beta_2 \rangle = \langle C_3\mathbf{k}, \beta_1 | \hat{H}^{\mathrm{DFT}}(C_3\mathbf{d}) | C_3\mathbf{k}, \beta_2 \rangle \tag{102}$$

and

$$E^{\mathrm{DFT}}_{\eta,\xi}(\mathbf{k}, \mathbf{d}) = E^{\mathrm{DFT}}_{\eta,\xi}(C_3\mathbf{k}, C_3\mathbf{d}) = E^{\mathrm{DFT}}_{\eta,\xi}(\mathbf{k}, C_3\mathbf{d}), \tag{103}$$

where the last line following from $E^{\mathrm{DFT}}_{\eta,\xi}$ depends on $|\mathbf{k}|$ in Eq. (101). So, only one of three stacking related by C_3 shown in Fig. 19(b)-(d) needs to be fitted.

Next we will establish the relation between the moiré Hamiltonian H_0 in Eq.1 of the main text and the Hamiltonian H^{DFT} constructed from the fitting to the DFT energy spectrum. We start from the momentum-space moiré Hamiltonian $\langle \mathbf{k}_1, \beta_1 | \hat{H}_0 | \mathbf{k}_2, \beta_2 \rangle$ in Eq.1 of the main text, where $\beta_{1,2} = 1, \cdots, 4$ label both layer and spin indices and

$|\mathbf{k},\beta\rangle$ are atomic Bloch states for each layer underlying the moiré superlattice. As the moiré Hamiltonian H_0 does not preserve atomic lattice translation, the crystal momentum $\mathbf{k}$ is not a good quantum number and H_0 can mix different $\mathbf{k}$ states. The atomic Bloch wave function $|\mathbf{k},\beta\rangle$ is related to atomic Wannier function $|\mathbf{R},\beta\rangle$ by

$$|\mathbf{k},\beta\rangle = \sum_{\mathbf{R}} e^{i\mathbf{k}\cdot\mathbf{R}}|\mathbf{R},\beta\rangle, \tag{104}$$

so the moiré Hamiltonian is transformed into the form on the atomic Wannier function basis as

$$\langle \mathbf{k}_1,\beta_1|\hat{H}_0|\mathbf{k}_2,\beta_2\rangle = \sum_{\mathbf{R}_1\mathbf{R}_2} e^{-i\mathbf{k}_1\cdot\mathbf{R}_1}\langle \mathbf{R}_1,\beta_1|\hat{H}_0|\mathbf{R}_2,\beta_2\rangle e^{i\mathbf{k}_2\cdot\mathbf{R}_2}. \tag{105}$$

Here $\langle \mathbf{R}_1,\beta_1|\hat{H}_0|\mathbf{R}_2,\beta_2\rangle$ describes the Hamiltonian matrix element between atomic Wannier functions located at $\mathbf{R}_1$ and $\mathbf{R}_2$ in the superlattice shown in Fig. 20(a). As the overlap between atomic Wannier functions decays quickly as the distance increases, we only consider the local hopping within the length scale $|\mathbf{R}_2-\mathbf{R}_1| \sim \mathcal{O}(|\tilde{\mathbf{a}}_1|)$, where $\tilde{\mathbf{a}}_1$ is the atomic primitive lattice vector for the Sb_2Te_3 layer. In this atomic length scale, the Hamiltonian matrix element between two Wannier orbitals near $\mathbf{R}$ on the superlattice structure with twist angle θ in Fig. 20(a) can be approximated locally by the Hamiltonian matrix element for two atomic layers with a constant shift $\mathbf{d}_{\mathbf{R}}$ in Fig. 20(b)[33], where

$$\mathbf{d}_{\mathbf{R}} = \mathcal{R}(\theta)\mathbf{R} - \mathbf{R} \tag{106}$$

and $\mathcal{R}(\theta)$ as the rotation operator for the Sb_2 layer with the rotating angle θ. This approximation is valid for a small twist angle θ because the local shift vector $\mathbf{d}_{\mathbf{R}}$ is almost uniform at the atomic length scale,

$$\mathbf{d}_{\mathbf{R}_2} \approx \mathbf{d}_{\mathbf{R}_1} \tag{107}$$

for $|\mathbf{R}_2-\mathbf{R}_1| \sim \mathcal{O}(|\tilde{\mathbf{a}}_1|)$. The Hamiltonian matrix element between two atomic Wannier orbitals for the commensurate lattice is captured by $\langle \mathbf{R}_1,\beta_1|\hat{H}^{\mathrm{DFT}}(\mathbf{d}_{\mathbf{R}_2})|\mathbf{R}_2,\beta_2\rangle$, so we make the approximation

$$\langle \mathbf{R}_1,\beta_1|\hat{H}_0|\mathbf{R}_2,\beta_2\rangle \approx \langle \mathbf{R}_1,\beta_1|\hat{H}^{\mathrm{DFT}}(\mathbf{d}_{\mathbf{R}_2})|\mathbf{R}_2,\beta_2\rangle \tag{108}$$

and

$$\langle \mathbf{k}_1,\beta_1|\hat{H}_0|\mathbf{k}_2,\beta_2\rangle = \sum_{\mathbf{R}_1\mathbf{R}_2} e^{-i\mathbf{k}_1\cdot\mathbf{R}_1}\langle \mathbf{R}_1,\beta_1|\hat{H}^{\mathrm{DFT}}(\mathbf{d}_{\mathbf{R}_2})|\mathbf{R}_2,\beta_2\rangle e^{i\mathbf{k}_2\cdot\mathbf{R}_2}. \tag{109}$$

To extract $\mathbf{R}_2$ in $\hat{H}^{\mathrm{DFT}}(\mathbf{d}_{\mathbf{R}_2})$ for the summation, we transform $\hat{H}^{\mathrm{DFT}}(\mathbf{d}_{\mathbf{R}})$ to the momentum-space by

$$\hat{H}^{\mathrm{DFT}}(\mathbf{d}_{\mathbf{R}}) = \sum_{\tilde{\mathbf{G}}} e^{-i\tilde{\mathbf{G}}\cdot\mathbf{d}_{\mathbf{R}}}\hat{H}^{\mathrm{DFT}}(\tilde{\mathbf{G}}) \tag{110}$$

as $\hat{H}^{\mathrm{DFT}}(\mathbf{d}_{\mathbf{R}} + x\tilde{\mathbf{a}}_1 + y\tilde{\mathbf{a}}_2) = \hat{H}^{\mathrm{DFT}}(\mathbf{d}_{\mathbf{R}})$ is periodic for atomic lattice vectors (x, y are integers here), as shown in Fig. 20(b). We also denote the atomic reciprocal lattice vector $\tilde{\mathbf{G}} = \tilde{\mathbf{G}}_{wz} = w\tilde{\mathbf{b}}_1 + z\tilde{\mathbf{b}}_2$ with integers w, z, so the summation over $\tilde{\mathbf{G}}$ is equivalent to the summation over w, z. $\tilde{\mathbf{b}}_{1,2}$ are atomic reciprocal lattice vectors satisfying $\tilde{\mathbf{b}}_i \cdot \tilde{\mathbf{a}}_j = \delta_{ij}$ for $i, j = 1, 2$ and shown in Fig. 20(c). Since

$$\tilde{\mathbf{G}}_{wz}\cdot\mathbf{d}_{\mathbf{R}} = \tilde{\mathbf{G}}_{wz}\cdot(\mathcal{R}(\theta)\mathbf{R}-\mathbf{R}) = (\tilde{\mathbf{G}}_{wz} - \mathcal{R}(\theta)\tilde{\mathbf{G}}_{wz})\cdot(\mathcal{R}(\theta)\mathbf{R}) = \mathbf{G}_{wz}\cdot(\mathbf{R}+\mathbf{d}_{\mathbf{R}}) \approx \mathbf{G}_{wz}\cdot\mathbf{R} \tag{111}$$

with the moiré reciprocal lattice vectors $\mathbf{G}_{wz}$ given by

$$\begin{aligned}\mathbf{G}_{wz} &= \tilde{\mathbf{G}}_{wz} - \mathcal{R}(\theta)\tilde{\mathbf{G}}_{wz}\\ &= (w\tilde{\mathbf{b}}_1 + z\tilde{\mathbf{b}}_2) - \mathcal{R}(\theta)(w\tilde{\mathbf{b}}_1 + z\tilde{\mathbf{b}}_2)\\ &= w(\tilde{\mathbf{b}}_1 - \mathcal{R}(\theta)\tilde{\mathbf{b}}_1) + z(\tilde{\mathbf{b}}_2 - \mathcal{R}(\theta)\tilde{\mathbf{b}}_2)\\ &= w(\tilde{\mathbf{b}}_1 - \tilde{\mathbf{b}}_1') + z(\tilde{\mathbf{b}}_2 - \tilde{\mathbf{b}}_2')\\ &= w\mathbf{b}_1^{\mathrm{M}} + z\mathbf{b}_2^{\mathrm{M}},\end{aligned} \tag{112}$$

we have

$$e^{-i\tilde{\mathbf{G}}\cdot\mathbf{d}_{\mathbf{R}}} \approx e^{-i\mathbf{G}\cdot\mathbf{R}}, \quad \hat{H}^{\mathrm{DFT}}(\mathbf{d}_{\mathbf{R}}) \approx \sum_{w,z} e^{-i\mathbf{G}_{wz}\cdot\mathbf{R}}\hat{H}^{\mathrm{DFT}}(\tilde{\mathbf{G}}_{wz}). \tag{113}$$

Here $\tilde{\mathbf{a}}'_{1,2} = \mathcal{R}(\theta)\tilde{\mathbf{a}}_{1,2}$ ($\tilde{\mathbf{b}}'_{1,2} = \mathcal{R}(\theta)\tilde{\mathbf{b}}_{1,2}$) are primitive (reciprocal) lattice vectors for the twisted Sb_2 layer as shown in Fig. 20(c) and $\mathbf{b}^{\mathrm{M}}_{1,2} = \tilde{\mathbf{b}}_{1,2} - \tilde{\mathbf{b}}'_{1,2}$ are the moiré reciprocal lattice vectors. The approximation in Eq. (111) is valid as $|\mathbf{d}_{\mathbf{R}}| \sim \mathcal{O}(|\tilde{\mathbf{a}}_1|) \ll |\mathbf{a}^{\mathrm{M}}_1|$ and $\mathbf{G} \cdot \mathbf{d}_{\mathbf{R}} \ll \mathbf{G} \cdot \mathbf{a}^{\mathrm{M}}_1 \sim \mathcal{O}(1)$.

Substituting Eq. (108) and (113) into Eq. (105) leads to

$$\begin{aligned}\langle \mathbf{k}_1, \beta_1 | \hat{H}_0 | \mathbf{k}_2, \beta_2 \rangle &\approx \sum_{\mathbf{R}_1 \mathbf{R}_2, w, z} e^{-i\mathbf{k}_1 \cdot \mathbf{R}_1} e^{-i\mathbf{G}_{wz} \cdot \mathbf{R}_2} \langle \mathbf{R}_1, \beta_1 | \hat{H}^{\mathrm{DFT}}(\tilde{\mathbf{G}}_{wz}) | \mathbf{R}_2, \beta_2 \rangle e^{i\mathbf{k}_2 \cdot \mathbf{R}_2} \\ &= \sum_{w,z} \langle \mathbf{k}_1, \beta_1 | \hat{H}^{\mathrm{DFT}}(\tilde{\mathbf{G}}_{wz}) | \mathbf{k}_2 - \mathbf{G}_{wz}, \beta_2 \rangle \\ &= \sum_{w,z} \delta(\mathbf{k}_2 - \mathbf{k}_1 - \mathbf{G}_{wz}) \langle \mathbf{k}_1, \beta_1 | \hat{H}^{\mathrm{DFT}}(\tilde{\mathbf{G}}_{wz}) | \mathbf{k}_1, \beta_2 \rangle. \end{aligned} \tag{114}$$

The last line comes from the conservation of crystal momenta of $H^{\mathrm{DFT}}(\mathbf{d})$,

$$\langle \mathbf{k}_1, \beta_1 | \hat{H}^{\mathrm{DFT}}(\tilde{\mathbf{G}}) | \mathbf{k}_2, \beta_2 \rangle = \int \mathrm{d}^2\mathbf{d} e^{i\tilde{\mathbf{G}} \cdot \mathbf{d}} \langle \mathbf{k}_1, \beta_1 | \hat{H}^{\mathrm{DFT}}(\mathbf{d}) | \mathbf{k}_2, \beta_2 \rangle = \delta(\mathbf{k}_1 - \mathbf{k}_2) \langle \mathbf{k}_1, \beta_1 | \hat{H}^{\mathrm{DFT}}(\tilde{\mathbf{G}}) | \mathbf{k}_1, \beta_2 \rangle. \tag{115}$$

Eq. (114) connects H^{DFT} and H_0 in atomic Bloch states in general, which is applied to the moiré potential in our model next.

We next show the relation between the potential $\tilde{\Delta}(\mathbf{d}_{\mathbf{R}})$ from DFT and the moiré potential $\Delta(\mathbf{r})$ in our model by Eq. (114). The Fourier transform of $\langle \mathbf{k}, \alpha | \hat{H}^{\mathrm{DFT}}(\mathbf{d}_{\mathbf{R}}) | \mathbf{k}, \alpha \rangle$ in Eq. (100) by Eq. (115) is

$$\langle \mathbf{k}, \beta_1 | \hat{H}^{\mathrm{DFT}}(\tilde{\mathbf{G}}) | \mathbf{k}, \beta_2 \rangle = \begin{pmatrix} h^t_D(\mathbf{k}) & m s_0 \\ m s_0 & h^b_D(\mathbf{k}) \end{pmatrix} \delta_{\tilde{\mathbf{G}}=0} + \begin{pmatrix} \tilde{\Delta}(\tilde{\mathbf{G}}) s_0 & 0 \\ 0 & \alpha \tilde{\Delta}(\tilde{\mathbf{G}}) s_0 \end{pmatrix}. \tag{116}$$

In atomic Bloch basis, the moiré Hamiltonian from Eq.1 of the main text without external electrical field is

$$\langle \mathbf{k}_1, \beta_1 | \hat{H}_0 | \mathbf{k}_2, \beta_2 \rangle = \begin{pmatrix} h^t_D(\mathbf{k}_1) & m s_0 \\ m s_0 & h^b_D(\mathbf{k}_1) \end{pmatrix} \delta(\mathbf{k}_2 - \mathbf{k}_1) + \begin{pmatrix} \Delta(\mathbf{G}) s_0 & 0 \\ 0 & \alpha \Delta(\mathbf{G}) s_0 \end{pmatrix} \delta(\mathbf{k}_2 - \mathbf{k}_1 - \mathbf{G}). \tag{117}$$

By comparison of two Hamiltonian following Eq. (114), one obtains

$$\Delta(\mathbf{G}_{wz}) = \tilde{\Delta}(\tilde{\mathbf{G}}_{wz}) \tag{118}$$

for w, z as integers, $\mathbf{G}_{wz} = w\mathbf{b}^{\mathrm{M}}_1 + z\mathbf{b}^{\mathrm{M}}_2$, and $\tilde{\mathbf{G}}_{wz} = w\tilde{\mathbf{b}}_1 + z\tilde{\mathbf{b}}_2$. In real space, this leads to

$$\Delta(\mathbf{R}) = \sum_{w,z} e^{-i\mathbf{G}_{w,z} \cdot \mathbf{R}} \Delta(\mathbf{G}_{wz}) \approx \sum_{w,z} e^{-i\tilde{\mathbf{G}}_{w,z} \cdot (\mathcal{R}(\theta)\mathbf{R} - \mathbf{R})} \tilde{\Delta}(\tilde{\mathbf{G}}_{wz}) = \tilde{\Delta}(\mathcal{R}(\theta)\mathbf{R} - \mathbf{R}) = \tilde{\Delta}(\mathbf{d}_{\mathbf{R}}) \tag{119}$$

following Eq. (111) and Eq. (118), reproducing Eq. (4) in the main text. It then can be interpolated to the whole real space by replacing the atomic lattice vectors $\mathbf{R}$ by the continuous variable $\mathbf{r}$,

$$\Delta(\mathbf{r}) \approx \tilde{\Delta}(\mathcal{R}(\theta)\mathbf{r} - \mathbf{r}), \tag{120}$$

because the atomic length scale is much smaller than the moiré length scale for small twist angles so that it is a good approximation to take the continuous limit for the atomic length scale.

Supplementary References

[1] Fu, L. & Kane, C. L. Topological insulators with inversion symmetry. *Physical Review B* **76**, 045302 (2007).
[2] Xu, Y. *et al.* High-throughput calculations of magnetic topological materials. *Nature* **586**, 702–707 (2020).
[3] Elcoro, L. *et al.* Magnetic topological quantum chemistry. *Nature communications* **12**, 1–10 (2021).
[4] Winkler, R. *Spin-orbit coupling effects in two-dimensional electron and hole systems*, vol. 191 (Springer, 2003).
[5] Kane, C. L. & Mele, E. J. Quantum spin hall effect in graphene. *Physical review letters* **95**, 226801 (2005).
[6] Pizzi, G. *et al.* Wannier90 as a community code: new features and applications. *Journal of Physics: Condensed Matter* **32**, 165902 (2020).
[7] Xu, Y. *et al.* Filling-enforced obstructed atomic insulators. *arXiv preprint arXiv:2106.10276* (2021).

[8] Zhang, Y., Jiang, K., Wang, Z. & Zhang, F. Correlated insulating phases of twisted bilayer graphene at commensurate filling fractions: A hartree-fock study. *Physical Review B* **102**, 035136 (2020).

[9] Lian, B. *et al.* Twisted bilayer graphene. iv. exact insulator ground states and phase diagram. *Physical Review B* **103**, 205414 (2021).

[10] Bernevig, B. A., Song, Z.-D., Regnault, N. & Lian, B. Twisted bilayer graphene. iii. interacting hamiltonian and exact symmetries. *Physical Review B* **103**, 205413 (2021).

[11] Liu, S., Khalaf, E., Lee, J. Y. & Vishwanath, A. Nematic topological semimetal and insulator in magic-angle bilayer graphene at charge neutrality. *Physical Review Research* **3**, 013033 (2021).

[12] Fu, L. Topological crystalline insulators. *Physical Review Letters* **106**, 106802 (2011).

[13] Fukui, T., Hatsugai, Y. & Suzuki, H. Chern numbers in discretized brillouin zone: efficient method of computing (spin) hall conductances. *Journal of the Physical Society of Japan* **74**, 1674–1677 (2005).

[14] Bouhon, A. *et al.* Non-abelian reciprocal braiding of weyl points and its manifestation in zrte. *Nature Physics* **16**, 1137–1143 (2020).

[15] Yu, J., Chen, Y.-A. & Sarma, S. D. Euler-obstructed cooper pairing: Nodal superconductivity and hinge majorana zero modes. *Physical Review B* **105**, 104515 (2022).

[16] Ahn, J., Park, S. & Yang, B.-J. Failure of nielsen-ninomiya theorem and fragile topology in two-dimensional systems with space-time inversion symmetry: application to twisted bilayer graphene at magic angle. *Physical Review X* **9**, 021013 (2019).

[17] Sorella, S. & Tosatti, E. Semi-metal-insulator transition of the hubbard model in the honeycomb lattice. *EPL (Europhysics Letters)* **19**, 699 (1992).

[18] Neto, A. C., Guinea, F., Peres, N. M., Novoselov, K. S. & Geim, A. K. The electronic properties of graphene. *Reviews of modern physics* **81**, 109 (2009).

[19] Guerci, D., Wang, J., Pixley, J. & Cano, J. Designer meron lattice on the surface of a topological insulator. *Physical Review B* **106**, 245417 (2022).

[20] Kresse, G. & Furthmüller, J. Efficient iterative schemes for ab initio total-energy calculations using a plane-wave basis set. *Physical review B* **54**, 11169 (1996).

[21] Perdew, J. P., Burke, K. & Ernzerhof, M. Generalized gradient approximation made simple. *Physical review letters* **77**, 3865 (1996).

[22] Blöchl, P. E. Projector augmented-wave method. *Physical review B* **50**, 17953 (1994).

[23] Kresse, G. & Joubert, D. From ultrasoft pseudopotentials to the projector augmented-wave method. *Physical review b* **59**, 1758 (1999).

[24] Grimme, S., Antony, J., Ehrlich, S. & Krieg, H. A consistent and accurate ab initio parametrization of density functional dispersion correction (dft-d) for the 94 elements h-pu. *The Journal of chemical physics* **132**, 154104 (2010).

[25] Liu, C.-X. *et al.* Model hamiltonian for topological insulators. *Physical Review B* **82**, 045122 (2010).

[26] Lucignano, P., Alfè, D., Cataudella, V., Ninno, D. & Cantele, G. Crucial role of atomic corrugation on the flat bands and energy gaps of twisted bilayer graphene at the magic angle $\theta \sim 1.08^{\circ}$. *Phys. Rev. B* **99**, 195419 (2019). URL `https://link.aps.org/doi/10.1103/PhysRevB.99.195419`.

[27] Uchida, K., Furuya, S., Iwata, J.-I. & Oshiyama, A. Atomic corrugation and electron localization due to moiré patterns in twisted bilayer graphenes. *Physical Review B* **90**, 155451 (2014).

[28] Koshino, M. *et al.* Maximally localized wannier orbitals and the extended hubbard model for twisted bilayer graphene. *Physical Review X* **8**, 031087 (2018).

[29] Kazmierczak, N. P. *et al.* Strain fields in twisted bilayer graphene. *Nature materials* **20**, 956–963 (2021).

[30] Enaldiev, V., Zolyomi, V., Yelgel, C., Magorrian, S. & Fal'Ko, V. Stacking domains and dislocation networks in marginally twisted bilayers of transition metal dichalcogenides. *Physical review letters* **124**, 206101 (2020).

[31] Vitek, V. Intrinsic stacking faults in body-centred cubic crystals. *Philosophical Magazine* **18**, 773–786 (1968).

[32] Zhou, S., Han, J., Dai, S., Sun, J. & Srolovitz, D. J. van der waals bilayer energetics: Generalized stacking-fault energy of graphene, boron nitride, and graphene/boron nitride bilayers. *Physical Review B* **92**, 155438 (2015).

[33] Jung, J., Raoux, A., Qiao, Z. & MacDonald, A. H. Ab initio theory of moiré superlattice bands in layered two-dimensional materials. *Physical Review B* **89**, 205414 (2014).
